# Adverse Events Reported with Standard-Dose and High-Dose Aflibercept: A FAERS Pharmacovigilance Study

**DOI:** 10.3390/vision10020018

**Published:** 2026-03-31

**Authors:** Minali Prasad, David J. Ramsey

**Affiliations:** 1 Chobanian & Avedisian School of Medicine, Boston University, Boston, MA 02118, USA; 2 Division of Ophthalmology, Department of Surgery, UMass Chan-Lahey School of Medicine, Burlington, MA 01805, USA; 3 Department of Ophthalmology, Tufts University School of Medicine, Boston, MA 02111, USA; 4 Department of Biomedical Sciences and Disease, New England College of Optometry, Boston, MA 02115, USA

**Keywords:** aflibercept, high-dose aflibercept, intravitreal injections, ocular adverse events, drug-related side effects and adverse reactions, pharmacovigilance

## Abstract

This pharmacovigilance study drew upon the U.S. Food and Drug Administration’s Adverse Event Reporting System (FAERS) database to compare the reporting patterns of ocular and systemic adverse events (AEs) for the 2 mg (standard-dose [SD]) and 8 mg (high-dose [HD]) formulations of aflibercept given for any ocular indication. Disproportionality analysis, including reporting odds ratios (ROR), was used to compare each dose individually to the background reporting rate for the AE. Statistical significance of the RORs was evaluated using Bonferroni correction, alongside signal detection based on Evans criteria, and Bayesian information components. The Breslow–Day test was used to conduct a head-to-head comparison of RORs between each dose. We identified 953 SD and 314 HD AE reports within the 750-day period after the approval of HD by the U.S. Food and Drug Administration (FDA; 8/18/2023). Compared to SD, HD had a higher ROR for endophthalmitis (HD: ROR 767.56 [95% CI, 466.11–1263.95]; SD: ROR 331.64 [95% CI, 216.71–507.51]), eye inflammation (HD: ROR 118.45 [95% CI, 55.85–251.20]; SD: 43.98 [95% CI, 21.87–88.44]), retinal vasculitis (HD: ROR 769.87 [95% CI, 337.13–1758.04]; SD: ROR 124.80 [95% CI, 39.67–392.63]), and systemic vasculitis (HD: ROR 28.40 [95% CI, 14.63–55.14]; SD: ROR 4.05 [1.52–10.82]). These results, based on FAERS, indicate associations rather than causal relationships. Further studies are needed to quantify the absolute risks and elucidate the mechanisms underlying differences in safety signals, if any.

## 1. Introduction

Intravitreal injection of medications targeting vascular endothelial growth factor (VEGF) has become the standard of care for retinal vascular diseases, including neovascular age-related macular degeneration (nAMD), diabetic macular edema (DME), and retinal vein occlusion (RVO). Widely used agents include aflibercept, ranibizumab, bevacizumab, brolucizumab, and faricimab. Rare but clinically important adverse events have been reported in both clinical trials and postmarketing surveillance, including intraocular inflammation, retinal vasculitis, endophthalmitis, and systemic vascular events [1,2,3,4,5].

Aflibercept is indicated for the treatment of nAMD, DME, diabetic retinopathy (DR), macular edema resulting from RVO, and retinopathy of prematurity [6]. Aflibercept was first approved by the U.S. Food and Drug Administration (FDA) on 18 November 2011 at a standard-dose (SD) formulation of 2 mg (Eylea^®^, Regeneron, Tarrytown, NY, USA), administered via intravitreal injection (0.05 mL of a 40 mg/mL solution) [7]. It has been shown to reduce macular edema and improve visual outcomes in several real-world clinical studies [3,8,9,10,11,12,13,14,15,16,17,18].

More recently, on 18 August 2023, a high-dose (HD) formulation of aflibercept at 8 mg (Eylea HD^®^, Regeneron Pharmaceuticals) was approved by the FDA for the treatment of nAMD, DME, and DR [19]. This formulation of aflibercept, also administered via intravitreal injection (0.07 mL of a 114.3 mg/mL solution), has been shown to maintain vision-based outcomes and anatomic measures of fluid control at extended dosing intervals [20,21,22]. These studies provide key evidence to support its durability, thereby reducing the treatment burden in real-world settings [23,24].

Notably, the PULSAR, PHOTON, and QUASAR phase 3 trials demonstrated no significant difference in ocular adverse drug reactions (ADRs) between SD aflibercept administered every 8 weeks and HD aflibercept administered up to every 12 weeks [20,21,22]. These trials included a total of 2559 individuals, of whom 804 were treated with SD, and 1755 were treated with HD. However, postmarketing safety characterization is necessary given that these large clinical trials are underpowered to detect rare ocular ADRs and are conducted using highly selected cohorts. Furthermore, real-world reporting patterns following the approval of a new formulation may differ from those in clinical trials. Therefore, the present pharmacovigilance study sought to compare the adverse event (AE) signals from SD and HD by using disproportionality metrics derived from the FDA Adverse Event Reporting System (FAERS) database.

## 2. Materials and Methods

### 2.1. FAERS Database

This study used the FAERS database (https://www.fda.gov/drugs/fdas-adverse-event-reporting-system-faers/fda-adverse-event-reporting-system-faers-public-dashboard [accessed 5 September 2025]), a publicly-accessible repository that comprises de-identified reports of AEs linked to medications [25]. FAERS serves as the FDA’s postmarketing safety surveillance program. This database, updated daily, contains reports of medication-associated AEs submitted by healthcare providers, consumers, or manufacturers from across the globe. Each report provides demographic information (age, sex, country where event occurred), drug information (suspected drug, active ingredient, indication), and AE information (reaction and outcomes). Unlike healthcare providers and consumers, manufacturers are required to submit AE reports. 

The study adhered to the principles of the Declaration of Helsinki and was deemed to meet the criteria for exempt human subjects research upon review by the Lahey Hospital & Medical Center Institutional Review Board. The requirement for informed consent was waived as it was a secondary analysis of existing health record data that had been de-identified in accordance with the HIPAA Privacy Rule (§164.514[a]).

### 2.2. Data Acquisition, Processing, and Eligibility of Reports

Quarterly FAERS data from 1 January 2004 to 5 September 2025 were accessed via the openFDA platform and imported into Visual Studio Code (version 1.106; Redmond, WA, USA) with Python (version 3.13.7) [26]. This information is organized into seven tables, including demographic information (DEMO), drug/biologic information (DRUG), AEs (REAC), patient outcomes (OUTC), report sources (RPSR), indications for medication (INDI), and dates of medication administration (THER) [27,28]. The DEMO, DRUG, and REAC tables were joined on the primary identification number into a master analysis table. Drug names were mapped to canonical names based on the United States Adopted Name Framework with Drugs@FDA. The AEs in the REAC table were standardized by using the Medical Dictionary for Regulatory Activities (MedDRA) preferred terms (PT).

Prior to data analysis, we performed single-value, within-case imputation on one variable of a demographic fingerprint that would be used later for de-duplication (age [rounded to the nearest whole month], sex, and country of report origin) if at least one prior report with the same case report number contained all demographic fields. Reports were then resolved based on the case report number, preferred term for the AE, and the demographic fingerprint. Incomplete reports were excluded if they were missing one or more of the following variables: age, sex, reporter country, PT, or case report number. If multiple reports with the same case report number, PT, and demographic fingerprint existed, then only the most recent version received by the FAERS was included in the analysis.

In the FAERS DRUG table, each medication reported in a case is assigned a role code indicating the assessment made by the reporter of its potential relationship to the adverse event, including designation as suspect (primary, secondary), concomitant, or interacting [27]. Only reports in which SD or HD was the primary suspect and administered for an ocular indication were included in the final analysis. We excluded cases where either formulation was the secondary suspect or listed as a concomitant or interacting drug. This was used to reduce the likelihood that the reported AE was more likely to be primarily attributed to another medication. Since FAERS reports may include multiple drugs, restricting the analysis to cases where SD or HD were identified as the primary suspect was employed to improve the specificity of signal detection. Cases with multiple AEs were included in the analyses of each AE category reported.

### 2.3. Classification of AE Reports

The primary analysis compared AE signals for SD and HD reported during a 750-day window beginning after the FDA approval of HD (18 August 2023 to 5 September 2025) [19]. Ocular and systemic AE reports were categorized into those ADRs listed in the prescribing information for either formulation of aflibercept, as well as additional ocular AEs reported in at least three unique cases. The analysis also evaluated AEs when grouped into Standardized MedDRA Queries (SMQs), predefined, validated groupings of MedDRA PTs.

We also conducted several key sensitivity analyses. First, we compared AEs for SD obtained from the first 750-day period after its approval by the FDA (18 November 2011 to 7 December 2013) to both HD and SD for AEs obtained from the 750-day window beginning after the FDA approval date for HD (the same period as above). This was in part to mitigate notoriety bias and eliminate the possibility of formulation misclassification since no HD was available in this period. We also compared SD-associated AE signals (listed in the package insert or not listed but mentioned in at least three unique cases for both dosages in the primary analysis) reported prior to approval of the prefilled syringe (12 August 2019) [29] with those reported after prefilled syringe approval through 5 September 2025.

### 2.4. Statistical Analysis

Statistical analyses were performed by using Python (version 3.13.7) [30]. The primary outcome was the reporting odds ratio (ROR). This was determined by means of a 2 × 2 contingency table, calculated as (a × d)/(b × c), where (a) is the number of reports for the AE of interest associated with the drug, (b) is the number of other AEs associated with the drug, (c) is the number of reports of the AE of interest with other drugs in the database, and (d) is the number of other AEs associated with other drugs [31]. The ROR is a measure of the likelihood of the AE to be associated with a drug relative to other drugs in the database. A 95% confidence interval was reported for the ROR. The Bonferroni correction was applied to the disproportionality analyses to reduce the risk of type 1 errors to account for multiple comparisons across the two formulations. We also used the criteria in Evans et al. to identify significant safety signals (at least 3 unique reports, a chi-squared statistic (χ^2^) > 4, and a proportional reporting ratio (PRR) > 2) [32]. We also calculated the lower bound of the 95% Bayesian credible interval for the information component (IC_025_) to further reduce the risk of detecting false-positive associations [31,33]. A positive IC_025_ indicates a statistically significant disproportionality signal. Statistical significance was defined by *p*-values of less than 0.05 (or lower, as required by the Bonferroni correction), positive IC_025_, and Evans criteria. 

When there were at least two formulation–AE associations that met statistical significance, we conducted a head-to-head comparison of the RORs between the formulations for the AE and indication, using the Breslow–Day test, a validated methodology that has been previously used to compare AE signals between brand, generic, and authorized generic drugs based on FAERS reporting [34]. All analyses were performed on the natural logarithm scale. A *p*-value of less than 0.05 for the Breslow–Day test was considered significant.

## 3. Results

### 3.1. Demographic Characteristics

**Figure 1:**
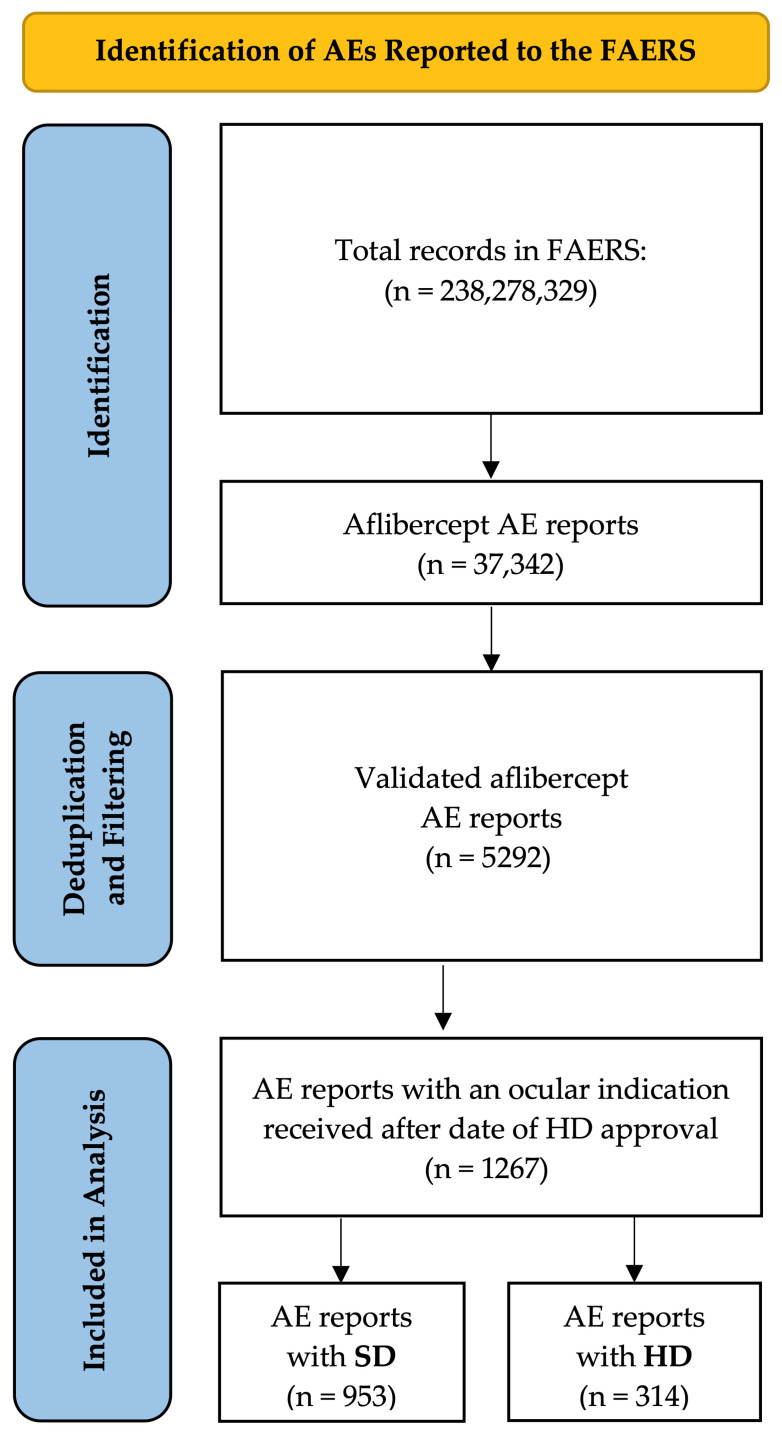
PRISMA diagram illustrating AE reports contained within the FAERS database for SD and HD within a 750-day study window beginning after the date of approval of HD by the FDA (18 August 2023). An irrelevant AE report was defined as one that did not meet the eligibility criteria for analysis. AE: adverse event; FDA: Food and Drug Administration; FAERS: FDA Adverse Event Reporting System; HD: high-dose aflibercept (8 mg); PRISMA: Preferred Reporting Items for Systematic Reviews and Meta-Analyses; SD: standard-dose aflibercept (2 mg).

A total of 953 SD and 314 HD reports met criteria for inclusion in the period after the approval of HD by the FDA [19] (Table 1 and Figure 1). Among SD reports, the majority involved males (51.6%), age 65–85 years (61.8%), and nAMD indication (54.6%). Among HD reports, the majority involved males (53.8%), age 65–85 years (71.7%), reporter source physician (65.6%), and nAMD indication (69.4%). There was no significant difference in the distribution of sex between SD and HD reports (*p* = 0.499). The distributions of age (*p* < 0.001), reporter continent (*p* < 0.001), and reporter source (*p* < 0.001) were significantly different between SD and HD reports.

### 3.2. Ocular AEs

**Figure 2:**
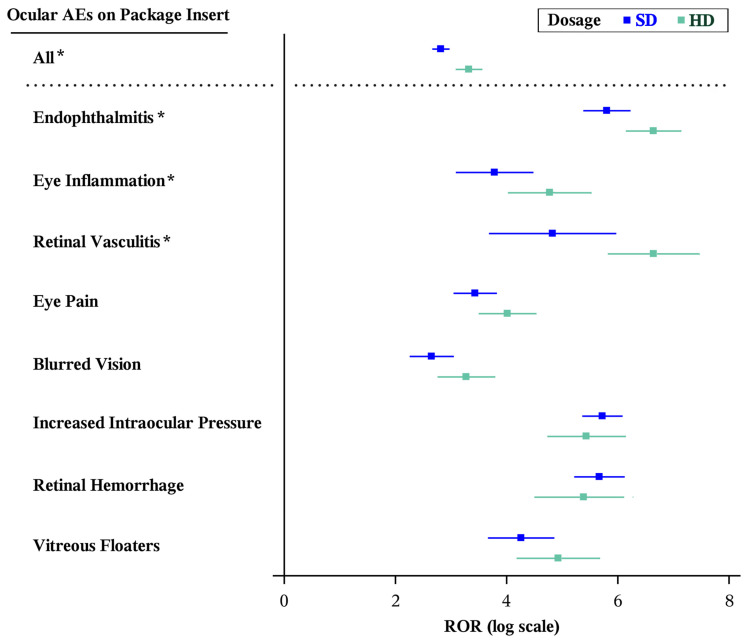
Forest plots of statistically significant (Bonferroni-corrected *p* < 0.0008, Evans criteria [n ≥ 3, χ^2^ > 4, PRR > 2], and IC_025_ > 0) disproportionality signals by ocular AEs found on the package insert of at least one aflibercept formulation. * Statistically significantly different RORs between formulations by the Breslow–Day test (*p* < 0.05). AE: adverse event; CI: confidence interval; HD: high-dose aflibercept (8 mg); IC: information component; PRR: proportional reporting ratio; ROR: reporting odds ratio; SD: standard-dose aflibercept (2 mg).

Compared to SD, HD had a significantly greater ROR for the group of all ocular ADRs listed on the package inserts (HD [*n* = 96]: ROR 27.75 [95% CI, 21.82–35.28]; SD [*n* = 200]: ROR 16.74 [95% CI, 14.32–19.56], *p* < 0.001). Several individual ocular AE categories individually met significance, including endophthalmitis (HD [*n* = 17]: ROR 767.56 [95% CI, 466.11–1263.95]; SD [*n* = 23]: ROR 331.64 [216.71–507.51], *p* = 0.009), eye inflammation (HD [*n* = 7]: ROR 118.45 [95% CI, 55.85–251.20]; SD [*n* = 8]: ROR 43.98 [95% CI, 21.87–88.44], *p* = 0.049), and retinal vasculitis (HD [*n* = 6]: ROR 769.87 [95% CI, 337.13–1758.04]; SD [*n* = 3]: ROR 124.80 [95% CI, 39.67–392.63], *p* = 0.004) (Table 2 and Figure 2). Of note, no reports of eyelid edema, corneal epithelium defects, lenticular opacities, foreign body sensation, hemorrhagic occlusive retinal vasculitis, or retinal pigment epitheliopathy were reported to FAERS within the study period. There were also no reports of retinal occlusive vasculitis or vitreous detachment for SD and no reports of retinal detachment, vitreous hemorrhage, increased lacrimation, conjunctival hemorrhage, detachment of macular retinal pigment epithelium, retinal pigment or epithelial tear for HD.

**Figure 3:**
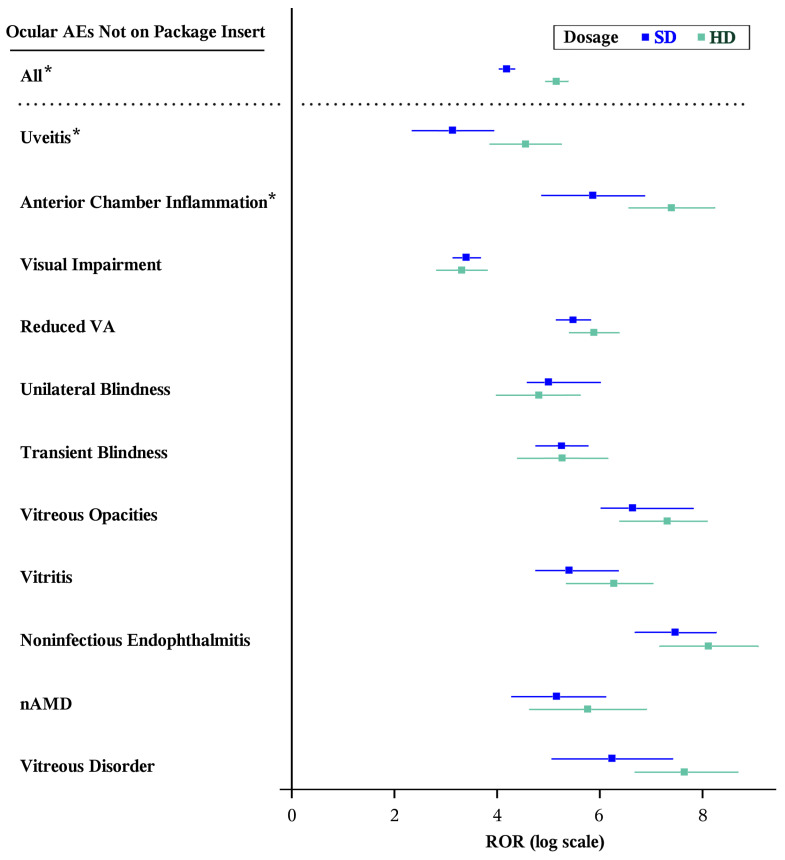
Forest plots of statistically significant (Bonferroni-corrected *p* < 0.001, Evans criteria [n ≥ 3, χ^2^ > 4, PRR > 2], and IC_025_ > 0) disproportionality signals by ocular AEs not found on the package inserts but reported in FAERS at least three times for either formulation of aflibercept. * Statistically significantly different RORs between formulations by the Breslow–Day test (*p* < 0.05). AE: adverse event; nAMD: age-related macular degeneration; CI: confidence interval; HD: high-dose aflibercept (8 mg); IC: information component; PRR: proportional reporting ratio; ROR: reporting odds ratio; SD: standard-dose aflibercept (2 mg); VA: visual acuity.

Compared to SD, HD also had a significantly greater ROR for all other ocular AEs identified in at least three reports to FAERS within the study period but for categories beyond those potential ADRs listed on the package inserts for either drug (HD [*n* = 122]: ROR 174.58 [95% CI, 139.07–219.14]; SD [*n* = 185]: ROR 66.18 [95% CI, 56.33–77.75], *p* < 0.001). In addition, the ROR for AEs for uveitis (HD [*n* = 8]: ROR 95.35 [95% CI, 47.17–192.75]; SD [*n* = 6]: ROR, 23.11 [95% CI, 10.34–51.65], *p* = 0.005) and anterior chamber inflammation (HD [*n* = 6]: ROR, 1639.09 [95% CI, 994.44–4879.14]; SD [*n* = 4]: ROR 354.65 [95% CI, 128.77–976.76], *p* = 0.013) met significance individually (Table 3 and Figure 3). There were no reports of keratitis for SD in the study period.

**Figure 4:**
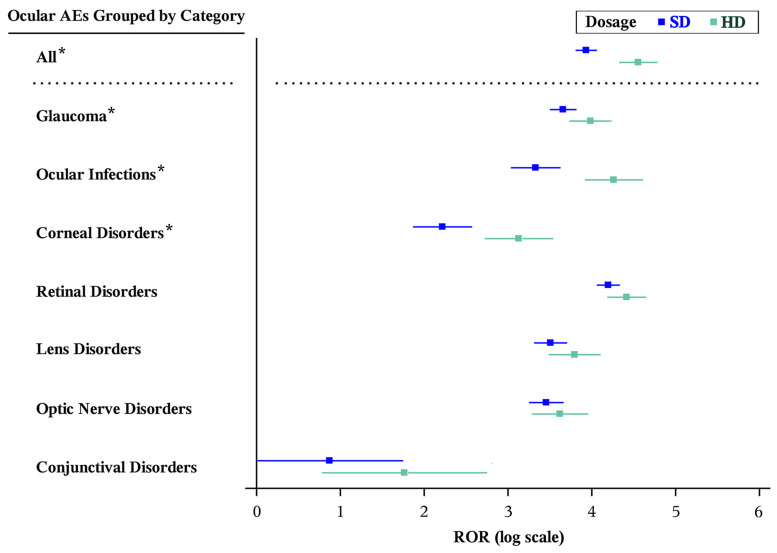
Forest plots of statistically significant (Bonferroni-corrected *p* < 0.002, Evans criteria [n ≥ 3, χ^2^ > 4, PRR > 2], and IC_025_ > 0) disproportionality signals of categories of ocular AEs included in the package insert by formulation of aflibercept. * Statistically significantly different RORs between formulations by the Breslow–Day test (*p* < 0.05). AE: adverse event; CI: confidence interval; HD: high-dose aflibercept (8 mg); IC: information component; PRR: proportional reporting ratio; SD: standard-dose aflibercept (2 mg); ROR: reporting odds ratio.

Compared to SD, HD also had a significantly greater ROR for ocular AEs grouped by category (HD [*n* = 195]: ROR 95.00 [95% CI, 75.63–119.34]; SD [*n* = 445]: ROR 51.03 [95% CI, 44.93–57.97], *p* < 0.001), as well as individually for corneal disorders (HD [*n* = 25]: ROR 22.86 [95% CI, 15.19–34.40]; SD [*n* = 32]: ROR 9.19 [95% CI, 6.46–13.07], *p* = 0.001), ocular infections (HD [*n* = 36]: ROR 70.96 [95% CI, 50.12–100.47]; SD [*n* = 46]: ROR 27.96 [95% CI, 20.78–37.63], *p* < 0.001), and glaucoma (HD [*n* = 81]: ROR 53.86 [95% CI, 41.82–69.36]; SD [*n* = 190]: ROR 38.80 [95% CI, 33.08–45.49], *p* < 0.001) (Table 4 and Figure 4). There were no reports of lacrimal disorders, periorbital and eyelid disorders, ocular motility disorders, or scleral disorders in the study period.

### 3.3. Systemic AEs

**Figure 5:**
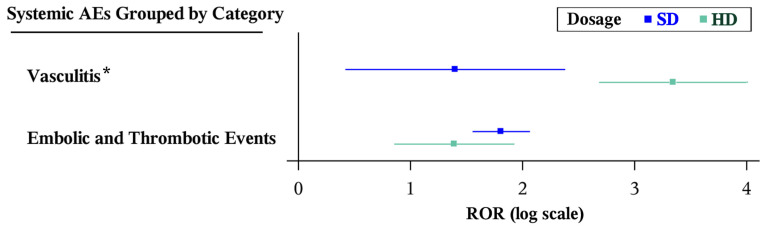
Forest plots of statistically significant (Bonferroni-corrected *p* < 0.003, Evans criteria [n ≥ 3, χ^2^ > 4, PRR > 2], and IC_025_ > 0) disproportionality signals of categories of systemic AEs (defined as an SMQ containing several PTs for AEs) included in the package insert by formulation of aflibercept. * Statistically significantly different RORs between formulations by the Breslow–Day test (*p* < 0.05). AE: adverse event; CI: confidence interval; HD: high-dose aflibercept (8 mg); IC: information component; PT: preferred term; PRR: proportional reporting ratio; SD: standard-dose aflibercept (2 mg); SMQ: Standard MedDRA Query; ROR: reporting odds ratio.

Compared to SD, HD had fewer total systemic AEs reported for potential ADRs listed on the label for each drug (Table 5). However, because no individual category of systemic AE had a significantly different ROR between the two formulations, no head-to-head comparison was performed by means of the Breslow–Day test. However, looking more broadly at systemic AEs beyond those listed on the package inserts identified a significantly greater ROR for systemic vasculitis for HD compared to SD (HD [*n* = 9]: ROR 28.40 [95% CI, 14.63–55.14]; SD [*n* = 4]: ROR 4.05 [95% CI, 1.52–10.82], *p* < 0.001) (Table 6 and Figure 5). There were no reports of anaphylactic reaction, anaphylactoid reaction, hypersensitivity, pruritis, urticaria, cardiac death, hemorrhagic stroke, accidental death, or sudden cardiac death for either formulation in the study period. There were no reports of sudden death for SD or myocardial infarction, cerebrovascular accident, or cardiac failure for HD.

### 3.4. Sensitivity Analyses of AEs

#### 3.4.1. AE Reports in the Period Immediately After SD FDA Approval vs. HD FDA Approval

After applying the same de-duplications and analysis criteria, a total of 335 AE reports linked to SD were identified in the first 750 days after SD approval (18 November 2011 to 7 December 2013) (Table S1). These were compared to the 314 HD reports in the first 750 days after the FDA approval of HD (18 August 2023 to 5 September 2025). The first ocular AE reported to FAERS for SD was endophthalmitis, reported 240 days (24 August 2012) after its FDA approval on 18 November 2011, and for HD, a case of blindness was reported 123 days (19 December 2023) after its FDA approval on 18 August 2023 [19]. Compared to SD in the period immediately after its FDA approval, HD had a higher ROR for ocular AEs for ADRs listed on the package insert, including a greater number of reports of increased intraocular pressure compared to SD (Table S2). HD also had more reported ocular AEs for other ADRs beyond those listed on the package inserts, including reduced visual acuity, visual impairment, and vitritis (Table S3). Compared to SD, HD also had more ocular AEs when grouped by category, including retinal disorders, glaucoma, lens disorders, and optic nerve disorders (Table S4). We could not conduct Breslow–Day tests with systemic AEs listed on the package insert or systemic AEs grouped by category because the signals did not meet Bonferroni correction, Evans criteria, or Bayesian IC thresholds (Tables S5 and S6).

#### 3.4.2. Comparison of AE Reports in the Period Immediately After FDA Approval of SD vs. More Recent Reports for SD After the FDA Approval of HD

A total of 335 SD reports during the initial 750 days after FDA approval were compared against 953 SD reports from 18 August 2023 to 5 September 2025, evaluated in the primary analysis. Compared to the initial period, the more recent period demonstrated a significantly lower ROR of eye inflammation, ocular hyperemia, and vitritis (Tables S8 and S9). Compared to the initial period, the more recent period demonstrated a significantly higher ROR of increased intraocular pressure, reduced VA, and visual impairment (Tables S7 and S8). Compared to the initial period, the more recent period demonstrated a significantly lower ROR of corneal disorders and ocular infections (Table S9). Compared to the initial period, the more recent period demonstrated a significantly higher ROR of retinal disorders and optic nerve disorders (Table S9). We could not conduct Breslow–Day tests for systemic AEs listed in the package insert or systemic AEs grouped by category because the signals did not meet Bonferroni correction, Evans criteria, or Bayesian IC thresholds (Tables S10 and S11).

#### 3.4.3. Ocular Infectious and Inflammatory AE Reports Pre- vs. Post-Approval of the SD Prefilled Syringe

A total of 1629 SD reports and 1741 SD reports before and after the approval of prefilled syringes (12 August 2019) were analyzed, respectively. Compared to the pre-prefilled syringe period, the post-prefilled syringe period had a significantly lower ROR of vitritis and eye inflammation (Table S12).

## 4. Discussion

In this large, pharmacovigilance study examining more than two years of real-world postmarketing AEs submitted to FAERS, we observed differences in the ROR for several ADRs listed on the package inserts for SD and HD. These included endophthalmitis, eye inflammation, and retinal vasculitis, as well as for inflammatory events not listed on either package insert, including uveitis and anterior chamber inflammation. SD was also associated with lower ROR for certain broad categories of ADRs, including glaucoma, corneal disorders, ocular infections, and retinal disorders. The analysis of most systemic AEs was limited by the low frequency of reports, precluding head-to-head comparisons of these RORs. HD had a stronger association with systemic vasculitis, but this was limited by a small case count yielding an ROR with a wide CI.

Importantly, many ADRs identified in large, randomized trials of both SD and HD were not identified in our study. During a more than two-year study period, there were no reports of corneal epithelium defects, lenticular opacities, hemorrhagic occlusive retinal vasculitis, or retinal pigment epitheliopathy. Although SD is approved for retinopathy of prematurity, our analysis identified no eligible reports with this indication.

A strength of our analysis is the selection of AE reports from an identical surveillance window for SD and HD, which began after the date of HD approval (18 August 2023). We also used Bayesian signal refinement and adjusted for the number of tests in each domain by applying Bonferroni-adjusted significance thresholds. Finally, we applied a rigorously defined de-duplication process linked to the report date to increase the measurement fidelity. We also de-duplicated by reporter country, and we excluded any reports without a documented ophthalmic indication. These differences in methodology likely account for the fewer AE reports evaluated by our study compared with a prior study that utilized some of the same data from FAERS to compare SD to HD [33]. Despite these differences, our results align with those of prior literature demonstrating higher RORs for HD for reduced VA, anterior chamber flare, toxic anterior segment syndrome, vitritis, retinal vasculitis, and infectious endophthalmitis compared to SD [33]. In contrast, our primary analysis demonstrated lower RORs for HD for increased intraocular pressure and retinal hemorrhage compared to SD, though these differences did not reach statistical significance [33].

Our postmarketing analysis study found that HD is associated with a higher ROR for AEs for intraocular inflammation, endophthalmitis, and retinal vasculitis compared with SD over a comparable period. While HD is labeled to allow for extended dosing intervals, which could reduce the frequency of its administration and associated treatment burdens [35,36,37], the higher-dose formulation could contribute to differences in ADR or reporting patterns. Notably, the higher volume administered was not linked to greater IOP-related AEs in clinical trials [20,21,22] or case series [23,24]. Our study also supports this finding through a review of post-marking surveillance data in FAERS.

Other variations between these two formulations of aflibercept may contribute to variations in the pattern of AEs identified by our study. There may be differences in the excipient between the HD and SD formulations. There may also be differences in the medication delivery technique, such as, different types of syringes or gauge needles used in real-world clinical practice [38]. Furthermore, many doses of SD are given by means of a prefilled syringe following its FDA approval [29]. However, these mechanisms are speculative and cannot be evaluated directly by means of FAERS data. We also are unable to take account of such factors as the level of experience of the individual who administered the medication, when in each patient’s treatment course the AE occurred, exposure history, or prior treatment with another dose or agent, if any. For example, prior studies have shown that the risk of endophthalmitis decreases as a patient receives more injections [39] and when medications are administered by more experienced providers, such as board-certified or retina-trained specialists [40]. We also lack data that allow for an understanding of patient-specific factors, past ocular history, and prior ocular treatment history. There is also the possibility that some AEs may represent misdiagnoses of non-infectious intraocular inflammation (sterile uveitis) as endophthalmitis, so our RORs need to be interpreted with appropriate caution. For example, an Intelligent Research in Sight (IRIS) Registry study demonstrated that a prior history of intraocular inflammation or RVO is a significant risk factor for an intraocular inflammation after an intravitreal injection of brolucizumab [41].

Further research is needed to optimize treatment outcomes in relation to injection burdens [42,43]. Although HD was found to have a similar safety profile compared with SD in large randomized clinical trials, including PULSAR and PHOTON [21] for nAMD and DME, respectively, those trials were underpowered to detect rare but serious AEs. The discrepancies between the clinical trial findings and our results are likely due to differences in study design. Both PULSAR and PHOTON [21] were randomized, double-masked, non-inferiority phase 3 and 2/3 trials, respectively. They standardized the injection frequency, technique, and follow-up period, selected for the ocular indication, and enrolled participants from only seven countries. Both studies were powered to demonstrate non-inferiority in best-corrected visual acuity and characterize common AEs. By contrast, the FAERS database captures reports from routine clinical practice across the globe for a variety of indications among a more heterogenous population, which allows for signal detection of rare events. While AEs were formally assessed during protocol visits in these studies, FAERS reports are reported by clinicians, the pharmaceutical industry, and patients. Such reports, most of which are voluntary, are subject to both under- and over-reporting of certain AEs [25].

ADRs listed in the package insert for SD and HD differ. Whereas both doses of the drug share in common a risk of cataract formation, conjunctival hemorrhage, corneal epithelium defects, endophthalmitis, eye pain, eyelid edema, injection site hemorrhage, eye inflammation, increased intraocular pressure, increased lacrimation, ocular hyperemia, retinal detachment, retinal tear, retinal vasculitis, blurred vision, vitreous detachment, vitreous floaters, detachment of macular retinal pigment epithelium, detachment of retinal pigment epithelium, hemorrhagic occlusive retinal vasculitis, retinal occlusive vasculitis, and retinal pigment epithelial tear, SD includes corneal edema, foreign body sensation, injection site pain, lenticular opacities, and scleritis, whereas HD includes eye irritation, retinal hemorrhage, vitreous hemorrhage, and retinal pigment epitheliopathy (Table S13). It is unknown if the difference in ADRs identified in the underlying pivotal Phase 3 trials differed between these two formulations because of dose or other factors.

The higher inflammatory and infectious RORs observed with HD in our study may have resulted for a variety of reasons. The HD formulation delivers a four-fold higher molar concentration of aflibercept, which may increase local drug load, alter tissue pharmacokinetics, and stimulate the immune response. Increased antigen exposure has been implicated in immune-mediated intraocular inflammation with other intravitreal biologics, including brolucizumab. Retinal vasculitis and intraocular inflammation were hypothesized to be triggered by the formation of immune complexes from anti-brolucizumab antibodies [44]. Furthermore, formulation differences from the excipients, silicone oil microdroplets produced by the intravitreal injection technique, or aggregates introduced during manufacturing, storage, or syringe handling can also contribute to intraocular inflammation [45,46,47]. However, it is important to note that we are unable to determine whether differences in the excipient or volume of medication administered account for differences in AEs. Although this study compares AEs between two formulations, the events may also be related to the injection itself. HD is available in a vial and must be transferred to the syringe by the provider, whereas SD is also available in a pre-filled syringe [29]. This may permit safer and more convenient handling of the medication. Our sensitivity analysis demonstrated significantly lower ROR for vitritis and eye inflammation after the approval of the prefilled SD syringe compared to before this date. Additionally, the selection of certain patients for HD, such as those who have treatment-resistant or more advanced stage disease, prior exposure to other anti-VEGF agents [24], or who have a greater underlying predisposition to inflammatory conditions, could lead to more inflammatory AEs independent of the formulation used, i.e., channeling bias.

We also explored selected systemic AE categories, focusing on vascular, cardiovascular, and immunologic events to reflect the type of systemic ADRs listed on the package inserts [48,49]. To our knowledge, this is the first postmarketing comparison of systemic AEs. In our study, we found no significant difference in signals between these formulations for central nervous system vascular disorders or embolic and thrombotic events. In fact, most other categories did not meet criteria for head-to-head comparison of ROR signals. We did find that HD had a stronger association with systemic vasculitis compared with SD, which may align with reports of systemic immune-mediated events associated with other potent, long-acting biologics in the eye [47]. Although systemic events after intravitreal anti-VEGF therapy are rare, case reports describe systemic inflammatory syndromes, delayed cutaneous hypersensitivity, angioedema, and Guillain–Barré syndrome following intravitreal medication [50,51,52,53]. While this signal may be influenced by channeling bias, concomitant medications, and comorbid autoimmune diseases, clinicians should be aware of systemic vasculitis as a potential rare adverse event warranting further investigation in patients receiving HD.

Because our study examined cases reported to FAERS in the period immediately after the approval of HD by the FDA, the rate of AE reports for this formulation may be heightened by increased physician awareness, recent regulatory scrutiny, or reporting stimulated by marketing or other media attention. We therefore performed a sensitivity analysis that compared AEs reported in the 750-day period immediately after the initial approval of SD by the FDA to the more contemporary AE reports (defined as the first 750 days after the approval of HD). This analysis found that the ROR of many ADRs listed on the package insert were lower for SD in the later, more recent period, including hyperemia, eye inflammation and vitritis, but they were higher for reduced VA, increased intraocular pressure, and visual impairment (Tables S7 and S8). By contrast, this analysis found a significantly higher ROR for increased intraocular pressure, reduced VA, visual impairment, vitritis, lens disorders, optic nerve disorders, glaucoma, and retinal disorders for HD compared with SD in a similar period immediately after initial approval (Tables S2–S4). This sensitivity analysis, similar to the primary analysis, demonstrated a significantly lower ROR of glaucoma for SD compared with HD, although with an even wider confidence interval. In contrast to the primary analysis, this sensitivity analysis failed to identify any difference in the AE signals for endophthalmitis, eye inflammation, retinal vasculitis, anterior chamber inflammation, uveitis, corneal disorders, ocular infections, or systemic vasculitis. Because FAERS does not capture changes in reporting practices, physician awareness, or postmarketing surveillance intensity over time, any difference in reporting patterns between these periods may represent residual reporting bias rather than a true difference in clinical risk.

Differences in case demographics or reporter characteristics between SD and HD may also have influenced the disproportionality signals observed in our study. We found that a greater proportion of SD versus HD reports were submitted to FAERS by consumers (38.7% vs. 6.7%). This leaves open the possibility that healthcare professionals were more likely to report significant inflammatory or infectious complications while under-reporting broader symptom-based complaints compared to consumers. The age distribution also differed between cases across formulations, with a greater proportion of HD reports occurring among older individuals. Since age is associated with both greater ocular disease severity and risk of complications, differences in disproportionality signals may reflect features of the underlying patient populations receiving each formulation rather than formulation-specific effects. Finally, in our study, HD reports were most commonly reported from Europe, whereas SD reports were most commonly reported from Asia. Consequently, the observed RORs may be influenced by differences in reporting behaviors across regions. Nevertheless, reporting of AEs to pharmacovigilance databases such as FAERS can facilitate signal detection and guide targeted investigations. 

Our study does not account for the frequency at which each formulation was prescribed. Within the study period, fewer doses of HD were likely given compared to SD [20,21,22,48,49,54,55]. Using 2024 Medicare Part B fee-for-service prescribing data, the most recent year available at the time of analysis, to estimate the rate at which SD and HD were administered yields an injection ratio of approximately 5:1, assuming that both formulations were utilized at this prevailing rate over the entire 750-day study period. This estimation does not capture doses given to individuals covered by Medicare Part C or D, Medicare Advantage, or those who had dual coverage. Injections delivered through non-Medicare systems such as those with commercial insurance, employer plans, Veterans Affairs or Indian Health Service, or who received injections as free drug samples, were also not accounted for. However, the Medicare Part B fee-for-service is the best publicly available source for estimating medication utilization. Although it is imprecise to extrapolate that HD was likely used less frequently on the basis of this subpopulation, the data, combined with HD’s recent approval, supports the conclusion that the higher AE rates for HD occurred despite fewer total doses likely administered. Future studies will also need to account for any changes in AE reports after the recent approval of HD for the treatment of RVO and the approval of a more frequent dosing regimen, when necessary [56].

The strengths of this study include its large sample size derived from a global population, its restriction to reports where aflibercept was given for an ocular indication and listed as the primary suspect by the reporter, and the use of multiple signal detection methods. We applied standard disproportionality techniques using the ROR and reduced false-positive signals after applying a Bonferroni correction, Evans criteria, and Bayesian information component thresholds. Head-to-head comparisons between formulations further strengthened our ability to detect true formulation- or dose-related differences beyond the relative differences reported in prior work [33]. Since FAERS reporting frequency, background drug use, and standards of care can change over time, the comparison of all-available SD reports with early HD reports can produce signals driven by calendar time and notoriety bias rather than dose. A further strength of our analysis is the use of periods restricted to the post-approval period for each drug to account for this bias.

Our study has several limitations inherent to the FAERS database. As outlined by the FDA, the existence of reports does not establish causation, the spontaneous nature of reporting precludes calculation of incidence rates or absolute risk, and the content of the reports themselves is not verified [57]. Without verified treatment exposure data, any potential causal relationship between the drug and a reported AE cannot therefore be adjudicated. Additionally, the database does not *require* the provision of drug formulation, indicating that some HD reports may have been misclassified and included in the SD analysis. However, given that SD is generally more commonly used than HD and is the only formulation available in certain countries based upon marketing authorization, reports with an unspecified aflibercept formulation most likely reflect SD. Furthermore, our study is limited in its ability to identify the specific version or manufacturer of the medication linked to an AE report, and only two reports specifically identified biosimilar versions of aflibercept (2 mg). We also restricted the analysis to reports in which SD or HD were designated as the primary suspect drug. While this improves the specificity of the analysis, it may have excluded cases in which aflibercept contributed to an ADR but was listed as a secondary suspect, concomitant, or interacting drug, potentially underestimating reporting signals. However, because aflibercept is most often used as monotherapy for retinal vascular disease, it is rarely listed as a secondary factor in most ophthalmic AE reports. Finally, although we report several large RORs for rare events, the precision of these estimates is quite limited despite meeting signal detection thresholds for significance. However, the direction of these disproportionality signals consistently pointed toward fewer safety concerns with SD despite substantially more injections of that formation likely administered in the study period.

In conclusion, fewer AEs were reported with SD compared to HD, but causal relationships between formulations and AEs cannot be established with FAERS data. SD had relatively fewer reports of intraocular inflammatory and infectious events, including endophthalmitis, uveitis, retinal vasculitis, and systemic vasculitis, compared with HD. Although clinicians should closely monitor all patients receiving intravitreal injections for inflammatory or infectious complications, this finding should inform future studies designed to quantify actual risk. In this postmarketing analysis, we emphasize that the observed differences in reporting patterns and distribution of ocular and systemic AEs are hypothesis-generating and not true safety signals, given the limitations of spontaneous reporting and limited number of reports. Future studies should use claims datasets to quantify absolute risks and identify patient-level predictors of AEs that may not be detectable in preapproval trials. As the number of individuals receiving anti-VEGF agents increases over time, there is a need for careful patient selection and mitigation strategies to reduce the risk of ADRs. In addition, reports of any suspected AEs should continue to be reported to the FAERS.

## Figures and Tables

**Table 1:** Demographic and clinical characteristics of patients with reported AEs for aflibercept, stratified by formulation.

Characteristic	Aflibercept 2 mg [SD] (*n* = 953)	Aflibercept 8 mg [HD] (*n* = 314)	*p*
Sex, No. (%)			0.499
Female	461 (48.4)	145 (46.2)	
Male	492 (51.6)	169 (53.8)	
Age, years, No. (%)			<0.001
<18	6 (0.6)	3 (1.0)	
18–44	29 (3.0)	3 (1.0)	
45–64	235 (24.7)	39 (12.4)	
65–85	589 (61.8)	225 (71.7)	
>85	94 (9.9)	44 (14.0)	
Continent, No. (%)			<0.001
North America	205 (21.5)	97 (30.9)	
South America	103 (10.8)	1 (0.3)	
Europe	275 (28.9)	153 (48.7)	
Asia	303 (31.8)	63 (20.1)	
Africa	16 (1.7)	0 (0)	
Oceania	51 (5.4)	0 (0)	
Reporter Source			<0.001
Physician	400 (42.0)	206 (65.6)	
Pharmacist	24 (2.5)	3 (1.0)	
Other health professional	160 (16.8)	84 (26.8)	
Consumer	369 (38.7)	21 (6.7)	
Indication, No. (%) ^§^			
nAMD	520 (54.6)	218 (69.4)	<0.001
RVO	60 (6.3)	9 (2.9)	
DME	211 (22.1)	67 (21.3)	0.766
DR	17 (1.8)	4 (1.3)	0.539
Other	145 (15.2)	16 (5.1)	

^§^ Only nAMD, DME, and DR were compared because these indications were approved for both formulations at the time of analysis. nAMD: neovascular age-related macular degeneration; DME: diabetic macular edema; DR: diabetic retinopathy; HD: high-dose aflibercept (8 mg); RVO: retinal vein occlusion; SD: standard-dose aflibercept (2 mg).

**Table 2:** Disproportionality analysis of ocular AEs reported to FAERS for ADRs listed on the package inserts.

Ocular AEs	Aflibercept 2 mg [SD]		Aflibercept 8 mg [HD]		Breslow-Day χ^2^	*p* ^†^
	No. (%)	ROR (95% CI)	No. (%)	ROR (95% CI)		
**Ocular** **AEs on** **Package Insert** ^§^	200 (21.0)	16.74 (14.32–19.56)	96 (30.6)	27.75 (21.82–35.28)	12.11	**<0.001**
Endophthalmitis	23 (2.4)	331.64 (216.71–507.51)	17 (5.4)	767.56 (466.11–1263.95)	6.65	**0.009**
Eye Inflammation	8 (0.8)	43.98 (21.87–88.44)	7 (2.2)	118.45 (55.85–251.20)	3.87	**0.049**
Retinal Vasculitis	3 (0.3)	124.80 (39.67–392.63)	6 (1.9)	769.87 (337.13–1758.04)	8.21	**0.004**
Eye Pain	26 (2.7)	31.02 (20.99–45.86)	15 (4.8)	55.49 (33.01–93.28)	3.15	0.076
Blurred Vision	25 (2.6)	14.19 (9.54–21.13)	15 (4.8)	26.43 (15.73–44.42)	3.58	0.059
Increased Intraocular Pressure	32 (3.4)	305.62 (212.90–438.73)	8 (2.5)	229.97 (113.45–466.15)	0.50	0.496
Retinal Hemorrhage	20 (2.1)	289.69 (183.97–456.16)	5 (1.6)	218.67 (89.87–532.08)	0.31	0.580
Vitreous Floaters	11 (1.2)	70.91 (38.99–128.96)	7 (2.2)	138.45 (65.26–293.74)	1.93	0.165
Cataract	9 (0.9)	9.81 (5.08–18.92)	1 (0.3)	3.29 (0.46–23.41)	— ^‡^	— ^‡^
Ocular Hyperemia	8 (0.8)	9.33 (4.65–18.71)	2 (0.6)	7.06 (1.76–28.37)	— ^‡^	— ^‡^
Injection Site Pain	7 (0.7)	1.09 (0.52–2.30)	1 (0.3)	0.47 (0.07–3.36)	— ^‡^	— ^‡^
Retinal Detachment	7 (0.7)	57.95 (27.45–122.37)	0 (0.0)	— ^¶^	— ^‡^	— ^‡^
Vitreous Hemorrhage	6 (0.6)	207.87 (91.77–470.84)	0 (0.0)	— ^¶^	— ^‡^	— ^‡^
Detachment of Retinal Pigment Epithelium	5 (0.5)	561.52 (223.24–1412.35)	1 (0.3)	340.14 (46.82–2470.93)	— ^‡^	— ^‡^
Eye Irritation	1 (0.1)	1.27 (0.18–9.05)	4 (1.3)	15.64 (5.83–41.95)	— ^‡^	— ^‡^
Corneal Edema	2 (0.2)	73.14 (18.10–295.58)	1 (0.3)	111.11 (15.50–796.45)	— ^‡^	— ^‡^
Retinal Tear	1 (0.1)	25.97 (3.64–185.41)	2 (0.6)	158.48 (39.20–640.68)	— ^‡^	— ^‡^
Retinal Occlusive Vasculitis	0 (0.0)	— ^¶^	3 (1.0)	852.91 (265.95–2735.28)	— ^‡^	— ^‡^
Increased Lacrimation	1 (0.1)	1.75 (0.25–12.47)	0 (0.0)	— ^¶^	— ^‡^	— ^‡^
Conjunctival Hemorrhage	1 (0.1)	26.99 (3.78–192.76)	0 (0.0)	— ^¶^	— ^‡^	— ^‡^
Injection Site Hemorrhage	1 (0.1)	0.66 (0.09–4.72)	0 (0.0)	— ^¶^	— ^‡^	— ^‡^
Scleritis	1 (0.1)	31.13 (4.36–222.49)	0 (0.0)	— ^¶^	— ^‡^	— ^‡^
Detachment of Macular Retinal Rigment Epithelium	1 (0.1)	913.30 (109.84–7593.55)	0 (0.0)	— ^¶^	— ^‡^	— ^‡^
Retinal Pigment Epithelial Tear	1 (0.1)	219.19 (29.67–1619.32)	0 (0.0)	325.24 (19.76–5354.29)	— ^‡^	— ^‡^
Vitreous Detachment	0 (0.0)	— ^¶^	1 (0.3)	99.20 (13.85–710.61)	— ^‡^	— ^‡^
Eyelid Edema	0 (0.0)	— ^¶^	0 (0.0)	— ^¶^	— ^‡^	— ^‡^
Corneal Epithelium Defect	0 (0.0)	— ^¶^	0 (0.0)	— ^¶^	— ^‡^	— ^‡^
Lenticular Opacities	0 (0.0)	— ^¶^	0 (0.0)	— ^¶^	— ^‡^	— ^‡^
Foreign Body Sensation	0 (0.0)	— ^¶^	0 (0.0)	— ^¶^	— ^‡^	— ^‡^
Hemorrhagic Occlusive Retinal Vasculitis	0 (0.0)	— ^¶^	0 (0.0)	— ^¶^	— ^‡^	— ^‡^
Retinal Pigment Epitheliopathy	0 (0.0)	— ^¶^	0 (0.0)	— ^¶^	— ^‡^	— ^‡^

^§^ Head-to-head comparisons for each AE that met the Bonferroni-corrected *p*-value (a *p* < 0.0008 threshold was applied to account for 62 comparisons across 31 ocular AEs listed on the package inserts and two formulations of aflibercept), Evans criteria (n ≥ 3, χ^2^ > 4, PRR > 2), and IC_025_ > 0 for both formulations of aflibercept were conducted with the Breslow–Day test. Formulation shaded in green represents AEs where the calculated ROR is statistically significantly lower using the Breslow–Day test. ^†^ Significance is marked in bold (*p* < 0.05). ^‡^ One or more formulations did not meet the Bonferroni correction threshold, Evans criteria, or IC_025_ > 0 for the AE, and therefore the Breslow–Day test could not be conducted. ^¶^ ROR: Not estimable because of a zero count AE in FAERS. AE: adverse event; CI: confidence interval; FAERS: Food and Drug Administration Adverse Event Reporting System; HD: high-dose aflibercept (8 mg); IC: information component; nAMD: age-related macular degeneration; PRR: proportional reporting ratio; ROR: reporting odds ratio; SD: standard-dose aflibercept (2 mg).

**Table 3:** Disproportionality analysis of ocular AEs reported to FAERS for ADRs not listed on the package inserts.

Ocular AEs	Aflibercept 2 mg [SD]		Aflibercept 8 mg [HD]		Breslow–Day χ^2^	*p* ^†^
	No. (%)	ROR (95% CI)	No. (%)	ROR (95% CI)		
**Additional Ocular AEs** **Not on** **Package Insert** ^§^	185 (19.4)	66.18 (56.33–77.75)	122 (38.9)	174.58 (139.07–219.14)	48.37	**<0.001**
Uveitis	6 (0.6)	23.11 (10.34–51.65)	8 (0.8)	95.35 (47.17–192.75)	7.91	**0.005**
Anterior Chamber Inflammation	4 (0.4)	354.65 (128.77–976.76)	6 (0.6)	1639.09 (703.80–3817.30)	6.17	**0.013**
Visual Impairment	53 (5.6)	30.20 (22.88–39.87)	16 (1.7)	27.53 (16.64–45.55)	0.10	0.753
Reduced VA	35 (3.7)	241.34 (171.03–340.56)	17 (1.8)	362.32 (221.19–593.52)	1.78	0.183
Unilateral Blindness	22 (2.3)	149.76 (97.59–229.83)	6 (0.6)	123.46 (54.88–277.75)	0.17	0.679
Transient Blindness	15 (1.6)	193.09 (114.94–324.38)	5 (0.5)	195.39 (80.35–475.13)	0.001	0.982
Vitreous Opacities	11 (1.2)	771.10 (409.04–1453.60)	7 (0.7)	1505.66 (689.52–3287.79)	1.77	0.183
Vitritis	9 (0.9)	224.03 (114.69–437.58)	7 (0.7)	535.78 (250.40–1146.42)	3.03	0.082
Non-Infectious Endophthalmitis	8 (0.8)	1766.51 (794.77–3926.35)	5 (0.5)	3376.52 (1284.27–8877.31)	1.08	0.300
Anterior Chamber Cell	2 (0.2)	203.17 (49.46–834.49)	7 (0.7)	2202.73 (994.44–4879.14)	— ^‡^	— ^‡^
Syringe Issue	1 (0.1)	1.94 (0.27–13.78)	7 (0.7)	42.08 (19.88–89.10)	— ^‡^	— ^‡^
Vitreal Cells	2 (0.2)	645.36 (148.89–2797.18)	6 (0.6)	5977.92 (2341.22–15263.61)	— ^‡^	— ^‡^
nAMD	5 (0.5)	175.25 (71.77–427.88)	3 (0.3)	320.51 (101.71–1009.98)	0.68	0.408
Vitreous Disorder	3 (0.3)	514.81 (157.37–1684.06)	4 (0.4)	2103.51 (739.50–5983.47)	3.54	0.060
Anterior Chamber Flare	2 (0.2)	498.68 (117.10–2123.72)	3 (0.3)	2287.37 (681.13–7681.45)	— ^‡^	— ^‡^
Ocular Discomfort	2 (0.2)	9.78 (2.44–39.20)	3 (0.3)	44.84 (14.36–140.00)	— ^‡^	— ^‡^
Metamorphopsia	2 (0.2)	92.97 (22.95–376.67)	3 (0.3)	426.45 (134.86–1348.54)	— ^‡^	— ^‡^
Ocular Hypertension	2 (0.2)	52.24 (12.96–210.57)	3 (0.3)	239.62 (76.25–753.04)	— ^‡^	— ^‡^
Toxic Anterior Segment Syndrome	1 (0.1)	161.17 (22.04–1178.60)	3 (0.3)	1480.06 (452.21–4844.18)	— ^‡^	— ^‡^
Keratitis	0 (0.0)	— ^¶^	3 (0.3)	181.01 (57.71–567.73)	— ^‡^	— ^‡^

^§^ Head-to-head comparisons for each AE that met the Bonferroni-corrected *p*-value (a *p* < 0.001 threshold was applied to account for 40 comparisons across 20 ocular AEs listed on the package inserts and two formulations of aflibercept), Evans criteria (n ≥ 3, χ^2^ > 4, PRR > 2), and IC_025_ > 0 for both formulations of aflibercept were conducted with the Breslow–Day test. Formulation shaded in green represents AEs where the calculated ROR is statistically significantly lower using the Breslow–Day test. ^†^ Significance is marked in bold (*p* < 0.05). ^‡^ One or more formulations did not meet the Bonferroni correction threshold, Evans criteria, or IC_025_ > 0 for the AE, and therefore the Breslow–Day test was not conducted. ^¶^ ROR: Not estimable because of a zero count AE in FAERS. AE: adverse event; CI: confidence interval; FAERS: Food and Drug Administration Adverse Event Reporting System; HD: high-dose aflibercept (8 mg); IC: information component; PRR: proportional reporting ratio; ROR: reporting odds ratio; SD: standard-dose aflibercept (2 mg); VA: visual acuity.

**Table 4:** Disproportionality analysis of ocular AEs reported to FAERS, grouped by SMQ.

	Aflibercept 2 mg [SD]		Aflibercept 8 mg [HD]		Breslow-Day χ^2^	*p* ^†^
	No. (%)	ROR (95% CI)	No. (%)	ROR (95% CI)		
**Ocular AEs Grouped by Category** ^§^	445 (46.7)	51.03 (44.93–57.97)	195 (62.1)	95.00 (75.63–119.34)	22.05	**<0.001**
Glaucoma	190 (19.9)	38.80 (33.08–45.49)	81 (25.8)	53.86 (41.82–69.36)	4.65	**0.031**
Ocular infections	46 (4.8)	27.96 (20.78–37.63)	36 (11.5)	70.96 (50.12–100.47)	16.89	**<0.001**
Corneal disorders	32 (3.4)	9.19 (6.46–13.07)	25 (8.0)	22.86 (15.19–34.40)	11.62	**0.001**
Retinal disorders	292 (30.6)	66.47 (57.90–76.32)	112 (35.7)	82.78 (65.69–104.30)	2.55	0.110
Lens disorders	113 (11.9)	33.36 (27.40–40.62)	48 (15.3)	44.51 (32.72–60.54)	2.41	0.121
Optic nerve disorders	102 (10.7)	31.73 (25.83–38.99)	39 (12.4)	37.34 (26.69–52.23)	0.66	0.418
Conjunctival disorders	5 (0.5)	2.39 (0.99–5.75)	4 (1.3)	5.84 (2.18–15.65)	1.88	0.170
Lacrimal disorders	4 (0.4)	2.08 (0.78–5.55)	0 (0.0)	— ^¶^	— ^‡^	— ^‡^
Periorbital and eyelid disorders	3 (0.3)	2.49 (0.80–7.73)	0 (0.0)	— ^¶^	— ^‡^	— ^‡^
Ocular motility disorders	2 (0.2)	4.36 (1.09–17.45)	0 (0.0)	— ^¶^	— ^‡^	— ^‡^
Scleral disorders	1 (0.10)	14.85 (2.08–105.80)	0 (0.0)	— ^¶^	— ^‡^	— ^‡^

^§^ Head-to-head comparisons for each AE that met the Bonferroni-corrected *p*-value (a *p* < 0.002 threshold was applied to account for 22 comparisons across 11 ocular AEs listed on the package inserts and two formulations of aflibercept), Evans criteria (n ≥ 3, χ^2^ > 4, PRR > 2), and IC_025_ > 0 for both formulations of aflibercept were conducted with the Breslow–Day test. Formulation shaded in green represents categories of AEs where the calculated ROR is statistically significantly lower using the Breslow–Day test. The total number of AEs includes ocular AEs listed in the package insert, those not listed in the package insert with at least three reports for either formulation, and those not listed in the package insert with fewer than 3 reports per dosage. ^†^ Significance is marked in bold (*p* < 0.05). ^‡^ One or more formulations did not meet the Bonferroni correction threshold, Evans criteria, or IC_025_ > 0 for the AE, and therefore the Breslow–Day test was not conducted. ^¶^ ROR: Not estimable because of a zero count AE in FAERS. AE: adverse event; CI: confidence interval; FAERS: Food and Drug Administration Adverse Event Reporting System; HD: high-dose aflibercept (8 mg); IC: information component; PRR: proportional reporting ratio; ROR: reporting odds ratio; SD: standard-dose aflibercept (2 mg).

**Table 5:** Disproportionality analysis of systemic AEs reported to FAERS for ADRs listed on the package inserts.

	Aflibercept 2 mg [SD]		Aflibercept 8 mg [HD]		Breslow-Day χ^2^	*p* ^†^
	No. (%)	ROR (95% CI)	No. (%)	ROR (95% CI)		
**Listed Systemic AEs from Package Insert** ^§^	11 (1.2)	0.43 (0.24–0.79)	2 (0.6)	0.24 (0.06–0.96)	— ^†^	— ^†^
Myocardial infarction	5 (0.5)	4.75 (1.97–11.44)	0 (0)	— ^¶^	— ^†^	— ^†^
Cerebrovascular accident	3 (0.3)	1.95 (0.63–6.07)	0 (0)	— ^¶^	— ^†^	— ^†^
Rash	2 (0.2)	0.28 (0.07–1.11)	1 (0.3)	0.42 (0.06–2.99)	— ^†^	— ^†^
Ischemic stroke	1 (0.1)	4.13 (0.58–29.34)	0 (0)	— ^¶^	— ^†^	— ^†^
Sudden death	0 (0)	— ^¶^	1 (0.3)	32.05 (4.49–228.67)	— ^†^	— ^†^
Anaphylactic reaction	0 (0)	0.47 (0.03–7.58)	0 (0)	— ^¶^	— ^†^	— ^†^
Anaphylactoid reaction	0 (0)	8.73 (0.54–139.91)	0 (0)	— ^¶^	— ^†^	— ^†^
Hypersensitivity	0 (0)	0.21 (0.01–3.29)	0 (0)	— ^¶^	— ^†^	— ^†^
Pruritis	0 (0)	0.06 (0.00–0.93)	0 (0)	— ^¶^	— ^†^	— ^†^
Urticaria	0 (0)	0.19 (0.01–2.98)	0 (0)	— ^¶^	— ^†^	— ^†^
Cardiac death	0 (0)	86.84 (5.31–1420.26)	0 (0)	— ^¶^	— ^†^	— ^†^
Hemorrhagic stroke	0 (0)	7.46 (0.47–119.64	0 (0)	— ^¶^	— ^†^	— ^†^
Accidental death	0 (0)	20.19 (1.26–324.67)	0 (0)	— ^¶^	— ^†^	— ^†^
Sudden cardiac death	0 (0)	24.10 (1.50–387.98)	0 (0)	— ^¶^	— ^†^	— ^†^

^§^ Head-to-head comparisons for each AE that met the Bonferroni-corrected *p*-value (a *p* < 0.002 threshold was applied to account for 28 comparisons across 14 ocular AEs listed on the package inserts and two formulations of aflibercept), Evans criteria (n ≥ 3, χ^2^ > 4, PRR > 2), and IC_025_ > 0 for both formulations of aflibercept were conducted with the Breslow–Day test. ^†^ One or more formulations did not meet the Bonferroni correction threshold, Evans criteria, or IC_025_ > 0 for the AE, and therefore the Breslow–Day test was not conducted. ^¶^ ROR: Not estimable because of a zero count AE in FAERS. AE: adverse event; CI: confidence interval; FAERS: Food and Drug Administration Adverse Event Reporting System; HD: high-dose aflibercept (8 mg); IC: information component; PRR: proportional reporting ratio; ROR: reporting odds ratio; SD: standard-dose aflibercept (2 mg).

**Table 6:** Disproportionality analysis of systemic AEs reported to FAERS, grouped by SMQ.

	Aflibercept 2 mg [SD]		Aflibercept 8 mg [HD]		Breslow-Day χ^2^	*p* ^†^
	No. (%)	ROR (95% CI)	No. (%)	ROR (95% CI)		
**Systemic AEs Grouped by Category** ^§^	108 (11.3)	0.73 (0.59–0.89)	34 (10.8)	0.69 (0.48–0.99)	— ^‡^	— ^‡^
Embolic and thrombotic events	63 (6.6)	6.10 (4.73–7.88)	14 (4.5)	4.02 (2.35–6.87)	1.93	0.165
Anaphylactic reaction	22 (2.3)	0.33 (0.21–0.50)	5 (1.6)	0.22 (0.09–0.54)	— ^‡^	— ^‡^
Central nervous system vascular disorders	18 (1.9)	2.84 (1.78–4.52)	6 (1.9)	2.87 (1.28–6.43)	— ^‡^	— ^‡^
Cardiomyopathy	12 (1.3)	0.45 (0.26–0.80)	3 (1.0)	0.34 (0.11–1.07)	— ^‡^	— ^‡^
Hypersensitivity	12 (1.2)	0.16 (0.09–0.29)	2 (0.6)	0.08 (0.02–0.33)	— ^‡^	— ^‡^
Vasculitis	4 (0.4)	4.05 (1.52–10.82)	9 (2.9)	28.40 (14.63–55.14)	13.93	**<0.001**
Ischemic Heart Diseases	7 (0.7)	1.91 (0.91–4.02)	1 (0.3)	0.82 (0.12–5.87)	— ^‡^	— ^‡^
Torsade de Pointes/QT Prolongation	4 (0.4)	0.51 (0.19–1.35)	3 (1.0)	1.16 (0.37–3.61)	— ^‡^	— ^‡^
Cardiac arrest	4 (0.4)	0.26 (0.10–0.68)	2 (0.6)	0.39 (0.10–1.56)	— ^‡^	— ^‡^
Cardiac failure	6 (0.6)	0.65 (0.29–1.45)	0 (0.0)	— ^¶^	— ^‡^	— ^‡^

^§^ Head-to-head comparisons for each AE that met the Bonferroni-corrected *p*-value (a *p* < 0.003 threshold was applied to account for 20 comparisons across 10 ocular AEs listed on the package inserts and two formulations of aflibercept), Evans criteria (n ≥ 3, χ^2^ > 4, PRR > 2), and IC_025_ > 0 for both formulations of aflibercept were conducted with the Breslow–Day test. Formulations shaded in green represent categories of AEs where the calculated ROR is statistically significantly lower using the Breslow–Day test. ^†^ Significance is marked in bold (*p* < 0.05). ^‡^ One or more formulations did not meet the Bonferroni correction threshold, Evans criteria, or IC_025_ > 0 for the AE, and therefore the Breslow–Day test was not conducted. ^¶^ ROR: Not estimable because of a zero count AE in FAERS. AE: adverse event; CI: confidence interval; FAERS: Food and Drug Administration Adverse Event Reporting System; HD: high-dose aflibercept (8 mg); IC: information component; PRR: proportional reporting ratio; ROR: reporting odds ratio; SD: standard-dose aflibercept (2 mg).

## Data Availability

The data analyzed in this study are available through the FDA Adverse Events Reporting System (https://open.fda.gov/data/downloads/ [accessed 5 September 2025]).

## Supplementary Materials

The following supporting information can be downloaded at https://www.mdpi.com/article/10.3390/vision10020018/s1, Table S1: Demographic and clinical characteristics of patients with reported AEs for aflibercept, stratified by formulation (SD data from 18 November 2011 to 7 December 2013, and HD data from 18 August 2023 to 5 September 2025); Table S2: Disproportionality analysis of ocular AEs reported to FAERS for ADRs listed on the package inserts (SD data from 18 November 2011 to 7 December 2013, and HD data from 18 August 2023 to 5 September 2025); Table S3. Disproportionality analysis of ocular AEs reported to FAERS for ADRs not listed on the package inserts (SD data from 18 November 2011 to 7 December 2013, and HD data from 18 August 2023 to 5 September 2025); Table S4: Disproportionality analysis of ocular AEs reported to FAERS, grouped by SMQ (SD data from 18 November 2011 to 7 December 2013, and HD data from 18 August 2023 to 5 September 2025); Table S5: Disproportionality analysis of systemic AEs reported to FAERS for ADRs listed on the package inserts (SD data from 18 November 2011 to 7 December 2013, and HD data from 18 August 2023 to 5 September 2025); Table S6: Disproportionality analysis of systemic AEs reported to FAERS, grouped by SMQ (SD data from 18 November 2011 to 7 December 2013, and HD data from 18 August 2023 to 5 September 2025); Table S7: Disproportionality analysis of ocular AEs reported to FAERS for SD as the primary suspect for ADRs listed on the package insert (initial data were collected from 18 November 2011 to 7 December 2013, and recent data from 18 August 2023 to 5 September 2025); Table S8: Disproportionality analysis of ocular AEs reported to FAERS with SD as the primary suspect for ADRs not listed on the package insert (initial data were collected from 18 November 2011 to 7 December 2013, and recent data from 18 August 2023 to 5 September 2025); Table S9: Disproportionality analysis of ocular AEs reported to FAERS with SD as the primary suspect, grouped by SMQ (initial data were collected from 18 November 2011 to 7 December 2013, and recent data from 18 August 2023 to 5 September 2025); Table S10: Disproportionality analysis of systemic AEs reported to FAERS with SD as the primary suspect for ADRs listed on the package inserts (initial data were collected from 18 November 2011 to 7 December 2013, and recent data from 18 August 2023 to 5 September 2025); Table S11: Disproportionality analysis of systemic AEs reported to FAERS with SD as the primary suspect, grouped by SMQ (initial data were collected from 18 November 2011 to 7 December 2013, and recent data from 18 August 2023 to 5 September 2025); Table S12: Disproportionality analysis of ocular infectious and inflammatory events for SD reports received before and after the approval of the prefilled syringe by the FDA (12 August 2019); Table S13: MedDRA preferred terms for the AEs mentioned on the package inserts.

## Abbreviations

The following abbreviations are used in this manuscript:

ADR	Adverse drug reaction
AE	Adverse events
Anti-VEGF	Anti-vascular endothelial growth factor
DME	Diabetic macular edema
DR	Diabetic retinopathy
FAERS	FDA Adverse Events Reporting System
FDA	Food & Drug Administration
HD	High-dose aflibercept (8 mg)
IC	Informational component
MedDRA	Medical Dictionary for Regulatory Activities
nAMD	Neovascular age-related macular degeneration
PRISMA	Preferred Reporting Items for Systematic Reviews and Meta-Analyses
PRR	Proportional reporting ratio
PT	Preferred term
ROR	Reporting odds ratio
RVO	Retinal vein occlusion
SD	Standard-dose aflibercept (2 mg)
SMQ	Standard MedDRA Query
