# Supplementary material for: Adverse Events Reported with Standard-Dose and High-Dose Aflibercept: A FAERS Pharmacovigilance Study
Source: Vision (Basel). 2026 Mar 31;10(2):18. doi: 10.3390/vision10020018 (PMC13108022; doi:10.3390/vision10020018)
Supplement: Supplementary file 1 [file vision-10-00018-s001.zip › vision-4180129-supplementary.pdf]

Supplementary Materials: Adverse Events Reported with Standard-Dose and High-Dose Aflibercept: A FAERS Pharmacovigilance Study

Minali Prasad and David J. Ramsey

Table S1. Demographic and clinical characteristics of patients with reported AEs for aflibercept, stratified by formulation (SD data from 18 November 2011 to 7 December 2013, and HD data from 18 August 2023 to 5 September 2025).

Characteristic	Aflibercept 2 mg [SD] (*n* = 335)	Aflibercept 8 mg [HD] (*n* = 314)	*p*
Sex, No. (%)			<0.001
Female	209 (62.4)	145 (46.2)	
Male	126 (37.6)	169 (53.8)	
Age, years, No. (%)			<0.001
<18	0 (0)	3 (1.0)	
18-44	1 (0.3)	3 (1.0)	
45-64	3 (0.9)	39 (12.4)	
65-85	258 (77.0)	225 (71.7)	
>85	73 (21.8)	44 (14.0)	
Continent, No. (%)			<0.001
North America	161 (48.1)	97 (30.9)	
South America	0 (0)	1 (0.3)	
Europe	168 (50.1)	153 (48.7)	
Asia	1 (0.3)	63 (20.1)	
Africa	0 (0)	0 (0)	
Oceania	5 (1.5)	0 (0)	
Reporter Source			<0.001
Physician	108 (32.2)	206 (65.6)	
Pharmacist	25 (7.5)	3 (1.0)	
Other Health Professional	126 (37.6)	84 (26.8)	
Consumer	17 (5.1)	21 (6.7)	
Unknown	59 (17.6)	0 (0)	
Indication, No. (%)[§]			
nAMD	324	218 (69.4)	<0.001
RVO	0	9 (2.9)	
DME	0	67 (21.3)	
DR	0	4 (1.3)	
Other	11	16 (5.1)	

[§] nAMD was the only indication approved by both formulations, and with a nonzero count for both formulations at the time of analysis. nAMD: neovascular age-related macular degeneration; DME: diabetic macular edema; DR: diabetic retinopathy; HD: high-dose aflibercept (8 mg); RVO: retinal vein occlusion: SD: standard-dose aflibercept (2 mg).

Table S2. Disproportionality analysis of ocular AEs reported to FAERS for ADRs listed on the package inserts (SD data from 18 November 2011 to 7 December 2013, and HD data from 18 August 2023 to 5 September 2025).

**Ocular AEs**	Aflibercept 2 mg [SD] (n=335)		Aflibercept 8 mg [HD] (n=314)		Breslow–Day χ^2	$p^{\dagger}$
	No. (%)	ROR (95% CI)	No. (%)	ROR (95% CI)		
**Ocular AEs Listed on Package Insert**[§]	79 (23.6)	18.52 (14.39 – 23.84)	96 (30.6)	27.75 (21.82 – 35.28)	5.20	**0.023**
Endophthalmitis	14 (4.2)	495.45 (284.04 – 864.20)	17 (5.4)	767.41 (466.02 – 1263.71)	1.34	0.247
Blurred Vision	14 (4.2)	17.64 (10.32 – 30.15)	15 (4.8)	26.43 (15.73 – 44.42)	1.14	0.285
Eye Pain	11 (3.3)	39.86 (21.81 – 72.84)	15 (4.8)	55.48 (33.00 – 93.26)	0.67	0.413
Retinal Hemorrhage	8 (2.4)	155.85 (76.58 – 317.18)	5 (1.6)	218.63 (89.85 – 531.97)	0.34	0.558
Eye Inflammation	5 (1.5)	167.09 (68.22 – 409.24)	7 (2.2)	118.43 (55.84 – 251.16)	0.336	0.562
Increased Intraocular Pressure	4 (1.2)	54.25 (20.15 – 146.08)	8 (2.5)	229.92 (113.43 – 466.05)	6.38	**0.012**
Vitreous Floaters	5 (1.5)	105.82 (43.40 – 257.98)	7 (2.2)	138.42 (65.24 – 293.68)	0.21	0.651
Ocular Hyperemia	6 (1.8)	27.43 (12.21 – 61.62)	2 (0.6)	7.06 (1.76 – 28.37)	—[‡]	—[‡]
Eye Irritation	2 (0.6)	10.03 (2.49 – 40.31)	4 (1.3)	15.63 (5.83 – 41.94)	—[‡]	—[‡]
Retinal Vasculitis	0 (0.0)	—[¶]	6 (1.9)	769.72 (337.07 – 1757.70)	—[‡]	—[‡]
Retinal Occlusive Vasculitis	0 (0.0)	—[¶]	3 (1.0)	852.75 (265.90 – 2734.74)	—[‡]	—[‡]
Injection Site Pain	1 (0.3)	0.41 (0.06 – 2.91)	1 (0.3)	0.47 (0.07 – 3.36)	—[‡]	—[‡]
Corneal Edema	1 (0.3)	99.06 (13.68 – 717.47)	1 (0.3)	111.09 (15.50 – 796.29)	—[‡]	—[‡]
Cataract	1 (0.3)	3.43 (0.48 – 24.42)	1 (0.3)	3.29 (0.46 – 23.41)	—[‡]	—[‡]
Retinal Tear	0 (0.0)	—[¶]	2 (0.6)	158.45 (39.19 – 640.55)	—[‡]	—[‡]
Retinal Pigment Epithelial Tear	2 (0.6)	353.96 (84.48 – 1482.95)	0 (0.0)	—[¶]	—[‡]	—[‡]
Detachment of Retinal Pigment Epithelium	1 (0.3)	176.45 (24.04 – 1295.05)	1 (0.3)	—[¶]	—[‡]	—[‡]
Foreign Body Sensation	1 (0.3)	32.26 (4.51 – 231.00)	0 (0.0)	—[¶]	—[‡]	—[‡]
Increased Lacrimation	1 (0.3)	7.24 (1.01 – 51.59)	0 (0.0)	—[¶]	—[‡]	—[‡]
Vitreous Detachment	0 (0.0)	—[¶]	1 (0.3)	99.19 (13.85 – 710.48)	—[‡]	—[‡]

Retinal Detachment	1 (0.3)	18.09 (2.53 – 129.24)	0 (0.0)	—¶	—‡	—‡
Retinal Pigment Epitheliopathy	1 (0.3)	1129.29 (131.58 – 9692.33)	0 (0.0)	—¶	—‡	—‡
Eyelid Edema	0 (0.0)	—¶	0 (0.0)	—¶	—‡	—‡
Conjunctival Hemorrhage	0 (0.0)	—¶	0 (0.0)	—¶	—‡	—‡
Injection Site Hemorrhage	0 (0.0)	—¶	0 (0.0)	—¶	—‡	—‡
Scleritis	0 (0.0)	—¶	0 (0.0)	—¶	—‡	—‡
Corneal Epithelium Defect	0 (0.0)	—¶	0 (0.0)	—¶	—‡	—‡
Lenticular Opacities	0 (0.0)	—¶	0 (0.0)	—¶	—‡	—‡
Vitreous Hemorrhage	0 (0.0)	—¶	0 (0.0)	—¶	—‡	—‡
Detachment of Macular Retinal Pigment Epithelium	0 (0.0)	—¶	0 (0.0)	—¶	—‡	—‡
Hemorrhagic Occlusive Retinal Vasculitis	0 (0.0)	—¶	0 (0.0)	—¶	—‡	—‡

§Head-to-head comparisons for each adverse event that met the Bonferroni-corrected *p-value* (a $p < 0.0008$ threshold was applied to account for 62 comparisons across 31 ocular AEs listed on the package inserts and two formulations of aflibercept), Evans criteria ($n \geq 3$, $\chi^2 > 4$, PRR > 2), and $IC_{025} > 0$ for both formulations of aflibercept were conducted with the Breslow–Day test. Formulation shaded in green represents AEs where the calculated ROR is statistically significantly lower using the Breslow–Day test. †Significance is marked in bold ($p < 0.05$). ‡One or more formulations did not meet the Bonferroni correction threshold, Evans criteria, or $IC_{025} > 0$ for the adverse event and therefore the Breslow–Day test was not conducted. ¶ROR: Not estimable because of a zero count AE in FAERS. AE: adverse event; CI: confidence interval; FAERS: Food and Drug Administration Adverse Event Reporting System; HD: high-dose aflibercept (8 mg); IC: information component; PRR: proportional reporting ratio; ROR: reporting odds ratio; SD: standard-dose aflibercept (2 mg).

Table S3. Disproportionality analysis of ocular AEs reported to FAERS for ADRs not listed on the package inserts (SD data from 18 November 2011 to 7 December 2013, and HD data from 18 August 2023 to 5 September 2025).

**Ocular AEs**	Aflibercept 2 mg [SD] (n=335)		Aflibercept 8 mg [HD] (n=314)		Breslow–Day χ^2	$p^{\dagger}$
	No. (%)	ROR (95% CI)	No. (%)	ROR (95% CI)		
**Additional Ocular AEs Not on Package Insert**[§]	66 (19.7)	69.65 (53.15 – 91.27)	122 (38.9)	174.58 (139.07 – 219.15)	26.70	**<0.001**
Reduced VA	24 (7.2)	84.34 (55.53 – 128.10)	17 (5.4)	362.25 (221.14 – 593.40)	23.28	**<0.001**
Visual Impairment	5 (1.5)	8.20 (3.39 – 19.84)	16 (5.1)	27.54 (16.65 – 45.55)	6.09	**0.014**
Vitritis	10 (3.0)	2000.94 (967.22 – 4139.44)	7 (2.2)	535.68 (250.35 – 1146.20)	6.49	**0.011**
Transient Blindness	5 (1.5)	138.03 (56.48 – 337.32)	5 (1.6)	195.35 (80.33 – 475.04)	0.30	0.587
Unilateral Blindness	4 (1.2)	56.40 (20.94 – 151.89)	6 (1.9)	123.59 (54.93 – 278.04)	1.52	0.218
Uveitis	2 (0.6)	36.65 (9.09 – 147.82)	8 (2.5)	95.33 (47.16 – 192.72)	—[‡]	—[‡]
Non-Infectious Endophthalmitis	5 (1.5)	1360.67 (510.03 – 3630.07)	5 (1.6)	3375.86 (1284.02 – 8875.58)	1.80	0.180
Vitreous Opacities	3 (0.9)	1549.21 (430.24 – 5578.34)	7 (2.2)	1505.36 (689.39 – 3287.16)	0.001	0.970
Anterior Chamber Inflammation	3 (0.9)	946.73 (277.55 – 3229.29)	6 (1.9)	1638.77 (703.67 – 3816.56)	—[‡]	—[‡]
Anterior Chamber Cell	1 (0.3)	352.90 (46.67 – 2668.71)	7 (2.2)	2202.30 (994.25 – 4878.19)	0.54	0.463
Syringe Issue	0 (0.0)	—[¶]	7 (2.2)	42.07 (19.87 – 89.08)	—[‡]	—[‡]
Vitreal Cells	1 (0.3)	16914.00 (687.76 – 415965.55)	6 (1.9)	5976.76 (2340.77 – 15260.65)	—[‡]	—[‡]
Keratitis	2 (0.6)	145.21 (35.53 – 593.40)	3 (1.0)	180.97 (57.70 – 567.62)	—[‡]	—[‡]
Vitreous Disorder	1 (0.3)	313.69 (41.76 – 2356.57)	4 (1.3)	2103.11 (739.36 – 5982.31)	—[‡]	—[‡]
Anterior Chamber Flare	0 (0.0)	—[¶]	3 (1.0)	2286.93 (681.00 – 7679.96)	—[‡]	—[‡]
Toxic Anterior Segment Syndrome	0 (0.0)	—[¶]	3 (1.0)	1479.77 (452.12 – 4843.24)	—[‡]	—[‡]
Ocular Hypertension	0 (0.0)	—[¶]	3 (1.0)	239.57 (76.23 – 752.90)	—[‡]	—[‡]
nAMD	0 (0.0)	—[¶]	3 (1.0)	320.45 (101.69 – 1009.79)	—[‡]	—[‡]

Ocular Discomfort	0 (0.0)	—[¶]	3 (1.0)	44.83 (14.36 – 139.98)	—[‡]	—[‡]
Metamorphopsia	0 (0.0)	—[¶]	3 (1.0)	426.37 (134.83 – 1348.28)	—[‡]	—[‡]

[§]Head-to-head comparisons for each adverse event that met the Bonferroni-corrected *p-value* (a $p < 0.001$ threshold was applied to account for 62 comparisons across 31 ocular AEs listed on the package inserts and two formulations of aflibercept), Evans criteria ($n \geq 3$, $\chi^2 > 4$, PRR > 2), and $IC_{025} > 0$ for both formulations of aflibercept were conducted with the Breslow–Day test. Formulation shaded in green represents AEs where the calculated ROR is statistically significantly lower using the Breslow–Day test. [†]Significance is marked in bold ($p < 0.05$). [‡]One or more formulations did not meet the Bonferroni correction threshold, Evans criteria, or $IC_{025} > 0$ for the adverse event and therefore the Breslow–Day test was not conducted. [¶]ROR: Not estimable because of a zero count AE in FAERS.AE: adverse event; CI: confidence interval; FAERS: Food and Drug Administration Adverse Event Reporting System; HD: high-dose aflibercept (8 mg); IC: information component; nAMD: neovascular age-related macular degeneration; PRR: proportional reporting ratio; ROR: reporting odds ratio; SD: standard-dose aflibercept (2 mg); VA: visual acuity.

Table S4. Disproportionality analysis of ocular AEs reported to FAERS, grouped by SMQ (SD data from 18 November 2011 to 7 December 2013, and HD data from 18 August 2023 to 5 September 2025).

**Ocular AEs**	Aflibercept 2 mg [SD] (n=335)		Aflibercept 8 mg [HD] (n=314)		Breslow–Day χ^2	$p^{\dagger}$
	No. (%)	ROR (95% CI)	No. (%)	ROR (95% CI)		
**Ocular AEs Grouped by Category**[§]	143 (42.7)	39.19 (31.55 – 48.67)	195 (62.1)	95.00 (75.63 – 119.34)	30.94	**<0.001**
Retinal Disorders	83 (24.8)	40.00 (31.20 – 51.28)	112 (35.7)	82.78 (65.69 – 104.30)	18.03	**<0.001**
Glaucoma	66 (19.7)	32.63 (24.92 – 42.74)	81 (25.8)	53.86 (41.82 – 69.36)	7.14	**0.008**
Lens Disorders	43 (12.8)	28.00 (20.32 – 38.60)	48 (15.3)	44.51 (32.72 – 60.54)	4.24	**0.040**
Optic Nerve Disorders	31 (9.3)	14.92 (10.30 – 21.60)	39 (12.4)	37.34 (26.69 – 52.23)	13.78	**<0.001**
Ocular Infections	24 (7.2)	61.17 (40.31 – 92.84)	36 (11.5)	70.96 (50.12 – 100.47)	0.29	0.592
Corneal Disorders	20 (6.0)	23.35 (14.84 – 36.72)	25 (8.0)	22.86 (15.19 – 34.40)	0.005	0.946
Conjunctival Disorders	3 (0.9)	8.22 (2.64 – 25.65)	4 (1.3)	5.84 (2.18 – 15.65)	0.20	0.653
Lacrimal Disorders	2 (0.6)	6.25 (1.56 – 25.12)	0 (0.0)	—[¶]	—[‡]	—[‡]
Periorbital and Eyelid Disorders	0 (0.0)	—[¶]	0 (0.0)	—[¶]	—[‡]	—[‡]
Scleral Disorders	0 (0.0)	—[¶]	0 (0.0)	—[¶]	—[‡]	—[‡]
Ocular Motility Disorders	0 (0.0)	—[¶]	0 (0.0)	—[¶]	—[‡]	—[‡]

[§]Head-to-head comparisons for each adverse event that met the Bonferroni-corrected *p-value* (a $p < 0.002$ threshold was applied to account for 22 comparisons across 11 ocular AEs listed on the package inserts and two formulations of aflibercept), Evans criteria (n ≥ 3, $\chi^2 > 4$, PRR > 2), and $IC_{025} > 0$ for both formulations of aflibercept were conducted with the Breslow–Day test. Formulation shaded in green represents categories of AEs where the calculated ROR is statistically significantly lower using the Breslow–Day test. The total number of AEs includes ocular AEs listed in the package insert, those not listed in the package insert with at least 3 reports for either formulation, and those not listed in the package insert with less than 3 reports per formulation. [†]Significance is marked in bold ($p < 0.05$). [‡]One or more formulations did not meet the Bonferroni correction threshold, Evans criteria, or $IC_{025} > 0$ for the adverse event and therefore the Breslow–Day test was not conducted. [¶]ROR: Not estimable because of a zero count AE in FAERS. AE: adverse event; CI: confidence interval; HD: high-dose aflibercept (8 mg); IC: information component; PT: preferred term; PRR: proportional reporting ratio; SD: standard-dose aflibercept (2 mg); SMQ: Standard MedDRA Query; ROR: reporting odds ratio.

Table S5. Disproportionality analysis of systemic AEs reported to FAERS for ADRs listed on the package inserts (SD data from 18 November 2011 to 7 December 2013, and HD data from 18 August 2023 to 5 September 2025).

**Systemic AEs**	Aflibercept 2 mg [SD] (n=335)		Aflibercept 8 mg [HD] (n=314)		Breslow–Day χ^2	*p*
	No. (%)	ROR (95% CI)	No. (%)	ROR (95% CI)		
**Listed Systemic AEs from Package Insert**[§]	4 (0.01)	0.44 (0.16 – 1.18)	2 (0.01)	0.24 (0.06 – 0.96)	—[†]	—[†]
Cerebrovascular Accident	2 (0.6)	1.97 (0.49 – 7.90)	0 (0.0)	—[¶]	—[†]	—[†]
Sudden Death	1 (0.3)	12.89 (1.81 – 91.97)	1 (0.3)	32.04 (4.49 – 228.62)	—[†]	—[†]
Rash	0 (0.0)	—[¶]	1 (0.3)	0.42 (0.06 – 2.99)	—[†]	—[†]
Myocardial Infarction	1 (0.3)	0.85 (0.12 – 6.06)	0 (0.0)	—[¶]	—[†]	—[†]
Anaphylactic Reaction	0 (0.0)	—[¶]	0 (0.0)	—[¶]	—[†]	—[†]
Anaphylactoid Reaction	0 (0.0)	—[¶]	0 (0.0)	—[¶]	—[†]	—[†]
Hypersensitivity	0 (0.0)	—[¶]	0 (0.0)	—[¶]	—[†]	—[†]
Pruritis	0 (0.0)	—[¶]	0 (0.0)	—[¶]	—[†]	—[†]
Urticaria	0 (0.0)	—[¶]	0 (0.0)	—[¶]	—[†]	—[†]
Cardiac Death	0 (0.0)	—[¶]	0 (0.0)	—[¶]	—[†]	—[†]
Ischemic Stroke	0 (0.0)	—[¶]	0 (0.0)	—[¶]	—[†]	—[†]
Hemorrhagic Stroke	0 (0.0)	—[¶]	0 (0.0)	—[¶]	—[†]	—[†]
Accidental Death	0 (0.0)	—[¶]	0 (0.0)	—[¶]	—[†]	—[†]
Sudden Cardiac Death	0 (0.0)	—[¶]	0 (0.0)	—[¶]	—[†]	—[†]

[§]Head-to-head comparisons for each adverse event that met the Bonferroni-corrected *p-value* (a $p < 0.002$ threshold was applied to account for 62 comparisons across 31 ocular AEs listed on the package inserts and two formulations of aflibercept), Evans criteria ($n \geq 3$, $\chi^2 > 4$, PRR > 2), and $IC_{025} > 0$ for both formulations of aflibercept were conducted with the Breslow–Day test. [†]One or more formulations did not meet the Bonferroni correction threshold, Evans criteria, or $IC_{025} > 0$ for the adverse event and therefore the Breslow–Day test was not conducted. [¶]ROR: Not estimable because of a zero count AE in FAERS. AE: adverse event; CI: confidence interval; FAERS: Food and Drug Administration Adverse Event Reporting System; HD: high-dose aflibercept (8 mg); IC: information component; PRR: proportional reporting ratio; ROR: reporting odds ratio; SD: standard-dose aflibercept (2 mg).

Table S6. Disproportionality analysis of systemic AEs reported to FAERS, grouped by SMQ (SD data from 18 November 2011 to 7 December 2013, and HD data from 18 August 2023 to 5 September 2025).

**Systemic AEs**	Aflibercept 2 mg [SD] (n=335)		Aflibercept 8 mg [HD] (n=314)		Breslow–Day χ^2	p
	No. (%)	ROR (95% CI)	No. (%)	ROR (95% CI)		
**Systemic AEs Grouped by Category**[§]	35 (10.4)	0.60 (0.42 – 0.85)	34 (10.8)	0.69 (0.48 – 0.99)	—[†]	—[†]
Anaphylactic Reaction	11 (3.3)	0.45 (0.25 – 0.82)	5 (1.6)	0.22 (0.09 – 0.54)	—[†]	—[†]
Cardiac Arrest	1 (0.3)	0.13 (0.02 – 0.91)	2 (0.6)	0.39 (0.10 – 1.56)	—[†]	—[†]
Cardiac Failure	0 (0.0)	—[¶]	0 (0.0)	—[¶]	—[†]	—[†]
Cardiomyopathy	5 (1.5)	0.41 (0.17 – 0.98)	3 (1.0)	0.34 (0.11 – 1.07)	—[†]	—[†]
Central Nervous System Vascular Disorders	7 (2.1)	1.84 (0.87 – 3.89)	6 (1.9)	2.87 (1.28 – 6.43)	—[†]	—[†]
Embolic and Thrombotic Events	13 (3.9)	1.74 (1.00 – 3.03)	14 (4.5)	4.02 (2.35 – 6.87)	—[†]	—[†]
Hypersensitivity	6 (1.8)	0.27 (0.12 – 0.61)	2 (0.6)	0.08 (0.02 – 0.33)	—[†]	—[†]
Ischemic Heart Diseases	3 (0.9)	1.03 (0.33 – 3.20)	1 (0.3)	0.82 (0.12 – 5.87)	—[†]	—[†]
Torsade de Pointes/QT Prolongation	1 (0.3)	0.30 (0.04 – 2.15)	3 (1.0)	1.16 (0.37 – 3.61)	—[†]	—[†]
Vasculitis	1 (0.3)	4.44 (0.62 – 31.62)	9 (2.9)	28.40 (14.63 – 55.14)	—[†]	—[†]

[§]Head-to-head comparisons for each adverse event that met Bonferroni-corrected *p-value* (a $p < 0.003$ threshold was applied to account for 20 comparisons across 10 ocular AEs listed on the package inserts and two formulations of aflibercept), Evans criteria (n ≥ 3, $\chi^2 > 4$, PRR > 2), and $IC_{025} > 0$ for both formulations were conducted with the Breslow–Day test. [†]One or more formulations did not meet the Bonferroni correction threshold, Evans criteria, or $IC_{025} > 0$ for the adverse event and therefore the Breslow–Day test was not conducted. [¶]ROR: Not estimable because of a zero count AE in FAERS. AE: adverse event; CI: confidence interval; FAERS: Food and Drug Administration Adverse Event Reporting System; HD: high-dose aflibercept (8 mg); IC: information component; PT: preferred term; PRR: proportional reporting ratio; ROR: reporting odds ratio; SD: standard-dose aflibercept (2 mg); SMQ: Standardized MedDRA Queries.

Table S7. Disproportionality analysis of ocular AEs reported to FAERS for SD as the primary suspect for ADRs listed on the package insert (initial data were collected from 18 November 2011 to 7 December 2013, and recent data from 18 August 2023 to 5 September 2025).

**Ocular AEs**	Aflibercept 2 mg [SD] Initial Period (n=335)		Aflibercept 2 mg [SD] More Recent Period (n=953)		Breslow–Day χ^2	$p^{\dagger}$
	No. (%)	ROR (95% CI)	No. (%)	ROR (95% CI)		
**Ocular AEs Listed on Package Insert**[§]	79 (23.6)	18.54 (14.40 – 23.86)	200 (21.0)	16.72 (14.31 – 19.55)	0.46	0.496
Endophthalmitis	14 (4.2)	530.49 (303.69 – 926.69)	23 (2.4)	329.02 (215.02 – 503.46)	1.81	0.179
Increased Intraocular Pressure	4 (1.2)	54.24 (20.14 – 146.05)	32 (3.4)	305.03 (212.49 – 437.87)	12.89	**<0.001**
Blurred Vision	14 (4.2)	17.65 (10.33 – 30.17)	25 (2.6)	14.17 (9.52 – 21.09)	0.42	0.518
Retinal Hemorrhage	8 (2.4)	157.95 (77.60 – 321.49)	20 (2.1)	288.87 (183.45 – 454.85)	2.02	0.155
Eyelid Edema	0 (0.0)	—[¶]	26 (2.7)	4.99 (0.31 – 79.87)	—[‡]	—[‡]
Vitreous Floaters	5 (1.5)	107.38 (44.04 – 261.83)	11 (1.2)	70.31 (38.66 – 127.88)	0.61	0.436
Ocular Hyperemia	6 (1.8)	27.42 (12.21 – 61.61)	8 (0.8)	9.32 (4.65 – 18.71)	4.31	**0.038**
Eye Pain	11 (3.3)	39.97 (21.87 – 73.05)	1 (0.1)	31.02 (20.99 – 45.86)	0.48	0.488
Eye Irritation	2 (0.6)	10.02 (2.49 – 40.30)	8 (0.8)	1.27 (0.18 – 9.05)	—[‡]	—[‡]
Cataract	1 (0.3)	3.43 (0.48 – 24.41)	9 (0.9)	9.81 (5.08 – 18.92)	—[‡]	—[‡]
Injection Site Pain	1 (0.3)	0.41 (0.06 – 2.91)	7 (0.7)	1.09 (0.52 – 2.30)	—[‡]	—[‡]
Retinal Detachment	1 (0.3)	18.27 (2.56 – 130.47)	7 (0.7)	58.03 (27.48 – 122.52)	—[‡]	—[‡]
Eye Inflammation	5 (1.5)	170.03 (69.41 – 416.55)	1 (0.1)	43.36 (21.56 – 87.20)	6.43	**0.011**
Vitreous Hemorrhage	0 (0.0)	—[¶]	6 (0.6)	207.82 (91.75 – 470.72)	—[‡]	—[‡]
Detachment of Retinal Pigment Epithelium	1 (0.3)	188.17 (25.59 – 1383.85)	5 (0.5)	561.38 (223.19 – 1412.00)	—[‡]	—[‡]
Corneal Edema	1 (0.3)	99.03 (13.67 – 717.30)	2 (0.2)	71.69 (17.74 – 289.66)	—[‡]	—[‡]
Retinal Vasculitis	0 (0.0)	—[¶]	3 (0.3)	118.49 (37.69 – 372.53)	—[‡]	—[‡]
Retinal Pigment Epithelial Tear	2 (0.6)	353.88 (84.46 – 1482.62)	1 (0.1)	238.19 (32.13 – 1765.58)	—[‡]	—[‡]
Increased Lacrimation	1 (0.3)	7.23 (1.01 – 51.57)	1 (0.1)	1.75 (0.25 – 12.48)	—[‡]	—[‡]

Foreign Body Sensation	1 (0.3)	32.26 (4.50 – 230.95)	0 (0.0)	—[¶]	—[‡]	—[‡]
Conjunctival Hemorrhage	0 (0.0)	—[¶]	1 (0.1)	26.99 (3.78 – 192.71)	—[‡]	—[‡]
Scleritis	0 (0.0)	—[¶]	1 (0.1)	31.48 (4.41 – 225.01)	—[‡]	—[‡]
Retinal Tear	0 (0.0)	—[¶]	1 (0.1)	25.72 (3.60 – 183.62)	—[‡]	—[‡]
Detachment of Macular Retinal Pigment Epithelium	0 (0.0)	—[¶]	1 (0.1)	913.07 (109.82 – 7591.67)	—[‡]	—[‡]
Retinal Pigment Epitheliopathy	1 (0.3)	1129.03 (131.55 – 9690.14)	0 (0.0)	—[¶]	—[‡]	—[‡]
Injection Site Hemorrhage	0 (0.0)	—[¶]	0 (0.0)	—[¶]	—[‡]	—[‡]
Corneal Epithelium Defect	0 (0.0)	—[¶]	0 (0.0)	—[¶]	—[‡]	—[‡]
Lenticular Opacities	0 (0.0)	—[¶]	0 (0.0)	—[¶]	—[‡]	—[‡]
Vitreous Detachment	0 (0.0)	—[¶]	0 (0.0)	—[¶]	—[‡]	—[‡]
Hemorrhagic Occlusive Retinal Vasculitis	0 (0.0)	—[¶]	0 (0.0)	—[¶]	—[‡]	—[‡]
Retinal Occlusive Vasculitis	0 (0.0)	—[¶]	0 (0.0)	—[¶]	—[‡]	—[‡]

[§]Head-to-head comparisons for each adverse event that met the Bonferroni-corrected *p-value* (a $p < 0.0008$ threshold was applied to account for 62 comparisons across 31 ocular AEs listed on the package inserts and two formulations of aflibercept), Evans criteria ($n \geq 3$, $\chi^2 > 4$, PRR > 2), and $IC_{025} > 0$ for both formulations were conducted with the Breslow–Day test. Formulation shaded in green represents AEs where the calculated ROR is statistically significantly lower using the Breslow–Day test. [†]Significance is marked in bold ($p < 0.05$). [‡]One or more formulations did not meet the Bonferroni correction threshold, Evans criteria, or $IC_{025} > 0$ for the adverse event and therefore the Breslow–Day test was not conducted. [¶]ROR: Not estimable because of a zero count AE in FAERS. AE: adverse event; CI: confidence interval; FAERS: Food and Drug Administration Adverse Event Reporting System; HD: high-dose aflibercept (8 mg); IC: information component; PRR: proportional reporting ratio; ROR: reporting odds ratio; SD: standard-dose aflibercept (2 mg).

Table S8. Disproportionality analysis of ocular AEs reported to FAERS with SD as the primary suspect for ADRs not listed on the package insert (initial data were collected from 18 November 2011 to 7 December 2013, and recent data from 18 August 2023 to 5 September 2025).

**Ocular AEs**	Aflibercept 2 mg [SD] Initial Period (n=335)		Aflibercept 2 mg [SD] More Recent Period (n=953)		Breslow–Day χ^2	$p^\dagger$
	No. (%)	ROR (95% CI)	No. (%)	ROR (95% CI)		
**Additional Ocular AEs Not on Package Insert**§	66 (19.7)	70.07 (53.47 – 91.82)	185 (19.4)	65.83 (56.03 – 77.34)	0.15	0.698
Reduced VA	24 (7.2)	85.16 (56.07 – 129.35)	35 (3.7)	239.82 (169.96 – 338.41)	15.37	**<0.001**
Visual Impairment	5 (1.5)	8.20 (3.39 – 19.84)	53 (5.6)	30.20 (22.88 – 39.87)	8.69	**0.003**
Unilateral Blindness	4 (1.2)	56.53 (20.99 – 152.24)	22 (2.3)	149.18 (97.21 – 228.93)	3.34	0.068
Transient Blindness	5 (1.5)	139.34 (57.01 – 340.57)	15 (1.6)	191.27 (113.87 – 321.30)	0.36	0.546
Vitritis	10 (3.0)	2417.26 (1146.77 – 5095.31)	9 (0.9)	216.18 (110.73 – 422.06)	31.24	**<0.001**
Vitreous Opacities	3 (0.9)	1548.86 (430.15 – 5577.08)	11 (1.2)	692.06 (368.57 – 1299.49)	1.28	0.259
Non-Infectious Endophthalmitis	5 (1.5)	1428.38 (532.92 – 3828.47)	8 (0.8)	1522.47 (694.21 – 3338.97)	0.01	0.921
Uveitis	2 (0.6)	37.74 (9.36 – 152.24)	6 (0.6)	23.12 (10.34 – 51.67)	—‡	—‡
Anterior Chamber Inflammation	3 (0.9)	1216.96 (348.11 – 4254.40)	4 (0.4)	323.27 (117.69 – 887.95)	2.91	0.088
nAMD	0 (0.0)	—¶	5 (0.5)	175.20 (71.76 – 427.78)	—‡	—‡
Vitreous Disorder	1 (0.3)	332.07 (44.06 – 2502.40)	3 (0.3)	445.13 (137.01 – 1446.16)	—‡	—‡
Keratitis	2 (0.6)	147.06 (35.98 – 601.11)	0 (0.0)	—¶	—‡	—‡
Anterior Chamber Cell	1 (0.3)	352.82 (46.66 – 2668.11)	2 (0.2)	161.30 (39.47 – 659.08)	—‡	—‡
Anterior Chamber Flare	0 (0.0)	—¶	2 (0.2)	406.23 (96.46 – 1710.73)	—‡	—‡
Ocular Hypertension	0 (0.0)	—¶	2 (0.2)	51.25 (12.72 – 206.56)	—‡	—‡
Vitreal Cells	1 (0.3)	16910.18 (687.60 – 415871.59)	2 (0.2)	353.82 (84.56 – 1480.49)	—‡	—‡
Ocular Discomfort	0 (0.0)	—¶	2 (0.2)	9.75 (2.43 – 39.08)	—‡	—‡
Metamorphopsia	0 (0.0)	—¶	2 (0.2)	90.65 (22.38 – 367.14)	—‡	—‡

Toxic Anterior Segment Syndrome	0 (0.0)	—[¶]	1 (0.1)	148.06 (20.29 – 1080.28)	—[‡]	—[‡]
Syringe Issue	0 (0.0)	—[¶]	1 (0.1)	1.93 (0.27 – 13.74)	—[‡]	—[‡]

[§]Head-to-head comparisons for each adverse event that met the Bonferroni-corrected *p-value* (a $p < 0.001$ threshold was applied to account for 40 comparisons across 20 ocular AEs listed on the package inserts and two formulations of aflibercept), Evans criteria ($n \geq 3$, $\chi^2 > 4$, PRR > 2), and $IC_{025} > 0$ for both formulations were conducted with the Breslow–Day test. Formulation shaded in green represents AEs where the calculated ROR is statistically significantly lower using the Breslow–Day test. [†]Significance is marked in bold ($p < 0.05$). [‡]One or more formulations did not meet the Bonferroni correction threshold, Evans criteria, or $IC_{025} > 0$ for the adverse event and therefore the Breslow–Day test was not conducted. [¶]ROR: Not estimable because of a zero count AE in FAERS. AE: adverse event; CI: confidence interval; FAERS: Food and Drug Administration Adverse Event Reporting System; HD: high-dose aflibercept (8 mg); IC: information component; nAMD: neovascular age-related macular degeneration; PRR: proportional reporting ratio; ROR: reporting odds ratio; SD: standard-dose aflibercept (2 mg); VA: visual acuity.

Table S9. Disproportionality analysis of ocular AEs reported to FAERS with SD as the primary suspect, grouped by SMQ (initial data were collected from 18 November 2011 to 7 December 2013, and recent data from 18 August 2023 to 5 September 2025).

**Ocular AEs**	Aflibercept 2 mg [SD] Initial Period (n=335)		Aflibercept 2 mg [SD] More Recent Period (n=953)		Breslow–Day χ^2	p[†]
	No. (%)	ROR (95% CI)	No. (%)	ROR (95% CI)		
**Ocular AEs Grouped by Category**[§]	143 (42.7)	39.19 (31.55 – 48.67)	445 (46.7)	51.03 (44.93 – 57.97)	4.25	**0.039**
Retinal Disorders	83 (24.8)	40.00 (31.20 – 51.28)	292 (30.6)	66.47 (57.90 – 76.32)	12.44	**<0.001**
Glaucoma	66 (19.7)	32.63 (24.92 – 42.74)	190 (19.9)	38.80 (33.08 – 45.49)	1.17	0.279
Lens Disorders	43 (12.8)	28.00 (20.32 – 38.60)	113 (11.9)	33.36 (27.40 – 40.62)	0.83	0.362
Optic Nerve Disorders	31 (9.3)	14.92 (10.30 – 21.60)	102 (10.7)	31.73 (25.83 – 38.99)	12.76	**<0.001**
Ocular Infections	24 (7.2)	61.17 (40.31 – 92.84)	46 (4.8)	27.96 (20.78 – 37.63)	9.41	**0.002**
Corneal Disorders	20 (6.0)	23.35 (14.84 – 36.72)	32 (3.4)	9.19 (6.46 – 13.07)	10.84	**<0.001**
Conjunctival Disorders	3 (0.9)	8.22 (2.64 – 25.65)	5 (0.5)	2.39 (0.99 – 5.75)	3.23	0.072
Lacrimal Disorders	2 (0.6)	6.25 (1.56 – 25.12)	4 (0.4)	2.08 (0.78 – 5.55)	—[‡]	—[‡]
Periorbital and Eyelid Disorders	0 (0.0)	—[¶]	3 (0.3)	2.49 (0.80 – 7.73)	—[‡]	—[‡]
Ocular Motility Disorders	0 (0.0)	—[¶]	2 (0.2)	4.36 (1.09 – 17.45)	—[‡]	—[‡]
Scleral Disorders	0 (0.0)	—[¶]	1 (0.10)	14.85 (2.08 – 105.80)	—[‡]	—[‡]

[§]Head-to-head comparisons for each adverse event that met the Bonferroni-corrected *p-value* (a $p < 0.002$ threshold was applied to account for 22 comparisons across 11 ocular AEs listed on the package inserts and two formulations of aflibercept), Evans criteria ($n \geq 3$, $\chi^2 > 4$, PRR > 2), and $IC_{025} > 0$ for both formulations of aflibercept were conducted with the Breslow–Day test. The total number of AEs includes ocular AEs listed in the package insert, those not listed in the package insert with at least 3 reports for either formulation, and those not listed in the package insert with less than 3 reports per formulation. [†]Significance is marked in bold ($p < 0.05$). [‡]One or more formulations did not meet the Bonferroni correction threshold, Evans criteria, or $IC_{025} > 0$ for the adverse event and therefore the Breslow–Day test was not conducted. [¶]ROR: Not estimable because of a zero count AE in FAERS. AE: adverse event; CI: confidence interval; HD: high-dose aflibercept (8 mg); IC: information component; PT: preferred term; PRR: proportional reporting ratio; SD: standard-dose aflibercept (2 mg); SMQ: Standard MedDRA Query; ROR: reporting odds ratio.

Table S10. Disproportionality analysis of systemic AEs reported to FAERS with SD as the primary suspect for ADRs listed on the package inserts (initial data were collected from 18 November 2011 to 7 December 2013, and recent data from 18 August 2023 to 5 September 2025).

Systemic AEs	Aflibercept 2 mg [SD] Initial Period (n=335)		Aflibercept 2 mg [SD] More Recent Period (n=953)		Breslow–Day χ^2	p
	No. (%)	ROR (95% CI)	No. (%)	ROR (95% CI)		
**Listed Systemic AEs from Package Insert**[§]	4 (0.01)	0.44 (0.16 – 1.18)	11 (1.2)	0.43 (0.24 – 0.79)	—[†]	—[†]
Myocardial Infarction	1 (0.3)	0.85 (0.12 – 6.06)	5 (0.5)	4.75 (1.97 – 11.44)	—[†]	—[†]
Cerebrovascular Accident	2 (0.6)	1.97 (0.49 – 7.91)	3 (0.3)	1.95 (0.63 – 6.07)	—[†]	—[†]
Rash	0 (0.0)	—[¶]	2 (0.2)	0.28 (0.07 – 1.11)	—[†]	—[†]
Ischemic Stroke	0 (0.0)	—[¶]	1 (0.1)	4.13 (0.58 – 29.35)	—[†]	—[†]
Sudden Death	1 (0.3)	12.89 (1.81 – 91.95)	0 (0.0)	—[¶]	—[†]	—[†]
Anaphylactic Reaction	0 (0.0)	—[¶]	0 (0.0)	—[¶]	—[†]	—[†]
Anaphylactoid Reaction	0 (0.0)	—[¶]	0 (0.0)	—[¶]	—[†]	—[†]
Hypersensitivity	0 (0.0)	—[¶]	0 (0.0)	—[¶]	—[†]	—[†]
Pruritis	0 (0.0)	—[¶]	0 (0.0)	—[¶]	—[†]	—[†]
Urticaria	0 (0.0)	—[¶]	0 (0.0)	—[¶]	—[†]	—[†]
Cardiac Death	0 (0.0)	—[¶]	0 (0.0)	—[¶]	—[†]	—[†]
Hemorrhagic Stroke	0 (0.0)	—[¶]	0 (0.0)	—[¶]	—[†]	—[†]
Accidental Death	0 (0.0)	—[¶]	0 (0.0)	—[¶]	—[†]	—[†]
Sudden Cardiac Death	0 (0.0)	—[¶]	0 (0.0)	—[¶]	—[†]	—[†]

[§]Head-to-head comparisons for each adverse event that met the Bonferroni-corrected *p-value* (a $p < 0.002$ threshold was applied to account for 28 comparisons across 14 ocular AEs listed on the package inserts and two formulations of aflibercept), Evans criteria (n ≥ 3, $\chi^2 > 4$, PRR > 2), and $IC_{025} > 0$ for both formulations of aflibercept were conducted with the Breslow–Day test. [†]One or more formulations did not meet the Bonferroni correction threshold, Evans criteria, or $IC_{025} > 0$ for the adverse event and therefore the Breslow–Day test was not conducted. AE: adverse event; CI: confidence interval; FAERS: Food and Drug Administration Adverse Event Reporting System; HD: high-dose aflibercept (8 mg); IC: information component; PRR: proportional reporting ratio; ROR: reporting odds ratio; SD: standard-dose aflibercept (2 mg).

Table S11. Disproportionality analysis of systemic AEs reported to FAERS with SD as the primary suspect, grouped by SMQ (initial data were collected from 18 November 2011 to 7 December 2013, and recent data from 18 August 2023 to 5 September 2025).

**Systemic AEs**	Aflibercept 2 mg [SD] Initial Period (n=335)		Aflibercept 2 mg [SD] More Recent Period (n=953)		Breslow–Day χ^2	p
	No. (%)	ROR (95% CI)	No. (%)	ROR (95% CI)		
**Systemic Aes Grouped by Category**[§]	35 (10.4)	0.60 (0.42 – 0.85)	108 (11.3)	0.73 (0.59 – 0.89)	—[†]	—[†]
Embolic and Thrombotic Events	13 (3.9)	1.74 (1.00 – 3.03)	63 (6.6)	6.10 (4.73 – 7.88)	—[†]	—[†]
Anaphylactic Reaction	11 (3.3)	0.45 (0.25 – 0.82)	22 (2.3)	0.33 (0.21 – 0.50)	—[†]	—[†]
Central Nervous System Vascular Disorders	7 (2.1)	1.84 (0.87 – 3.89)	18 (1.9)	2.84 (1.78 – 4.52)	—[†]	—[†]
Hypersensitivity	6 (1.8)	0.27 (0.12 – 0.61)	12 (1.2)	0.16 (0.09 – 0.29)	—[†]	—[†]
Cardiomyopathy	5 (1.5)	0.41 (0.17 – 0.98)	12 (1.3)	0.45 (0.26 – 0.80)	—[†]	—[†]
Ischemic Heart Diseases	3 (0.9)	1.03 (0.33 – 3.20)	7 (0.7)	1.91 (0.91 – 4.02)	—[†]	—[†]
Cardiac Failure	0 (0.0)	—[¶]	6 (0.6)	0.65 (0.29 – 1.45)	—[†]	—[†]
Cardiac Arrest	1 (0.3)	0.13 (0.02 – 0.91)	4 (0.4)	0.26 (0.10 – 0.68)	—[†]	—[†]
Torsade de Pointes/QT Prolongation	1 (0.3)	0.30 (0.04 – 2.15)	4 (0.4)	0.51 (0.19 – 1.35)	—[†]	—[†]
Vasculitis	1 (0.3)	4.44 (0.62 – 31.62)	4 (0.4)	4.05 (1.52 – 10.82)	—[†]	—[†]

[§]Head-to-head comparisons for each adverse event that met Bonferroni-corrected *p-value* (a $p < 0.003$ threshold was applied to account for 20 comparisons across 10 ocular AEs listed on the package inserts and two formulations of aflibercept), Evans criteria (n ≥ 3, $\chi^2 > 4$, PRR > 2), and $IC_{025} > 0$ for both formulations were conducted with the Breslow–Day test. [†]One or more formulations did not meet the Bonferroni correction threshold, Evans criteria, or $IC_{025} > 0$ for the adverse event and therefore the Breslow–Day test was not conducted. [¶]ROR: Not estimable because of a zero count AE in FAERS. AE: adverse event; CI: confidence interval; FAERS: Food and Drug Administration Adverse Event Reporting System; HD: high-dose aflibercept (8 mg); IC: information component; PT: preferred term; PRR: proportional reporting ratio; ROR: reporting odds ratio; SD: standard-dose aflibercept (2 mg); SMQ: Standardized MedDRA Queries.

Table S12. Disproportionality analysis of ocular infectious and inflammatory events for SD reports received before and after the approval of the prefilled syringe by the FDA (12 August 2019).

**Endophthalmitis AEs**	Aflibercept 2 mg [SD] Preapproval (n=1629)		Aflibercept 2 mg [SD] Postapproval (n=1741)		Breslow–Day χ^2	$p^{\dagger}$
	No. (%)	ROR (95% CI)	No. (%)	ROR (95% CI)		
**Ocular Infectious and Inflammatory AEs**[§]	173 (10.6)	277.70 (236.72 – 325.76)	125 (7.2)	110.78 (92.26 – 133.01)	58.34	**<0.001**
Keratitis	3 (0.18)	37.09 (11.92 – 115.40)	0 (0.00)	5.90 (0.37 – 94.48)	—‡	—‡
Scleritis	0 (0.00)	14.58 (0.91 – 233.72)	1 (0.06)	16.94 (2.38 – 120.57)	—‡	—‡
Anterior Chamber Inflammation	5 (0.31)	374.58 (152.79 – 918.29)	7 (0.40)	314.40 (147.76 – 668.96)	0.09	0.769
Endophthalmitis	60 (3.68)	543.71 (417.01 – 708.89)	54 (3.10)	430.45 (326.31 – 567.83)	1.43	0.232
Non-Infectious Endophthalmitis	12 (0.74)	1207.34 (658.67 – 2213.06)	12 (0.69)	869.17 (479.78 – 1574.59)	0.58	0.446
Vitritis	29 (1.78)	1249.21 (843.96 – 1849.06)	16 (0.92)	249.04 (151.22 – 410.16)	29.45	**<0.001**
Retinal Vasculitis	1 (0.06)	45.47 (6.37 – 324.69)	4 (0.23)	77.19 (28.82 – 206.76)	—‡	—‡
Retinal Occlusive Vasculitis	0 (0.00)	—¶	1 (0.06)	—¶	—‡	—‡
Hemorrhagic Occlusive Retinal Vasculitis	0 (0.00)	—¶	0 (0.00)	—¶	—‡	—‡
Uveitis	10 (0.61)	40.68 (21.81 – 75.86)	11 (0.63)	23.14 (12.78 – 41.90)	1.69	0.193
Eye Inflammation	53 (3.25)	363.87 (275.25 – 481.00)	19 (1.09)	64.73 (41.12 – 101.89)	50.34	**<0.001**

§Head-to-head comparisons for each adverse event that met Bonferroni-corrected *p-value* (a $p < 0.002$ threshold was applied to account for 22 comparisons across 11 ocular AEs listed on the package inserts and two time periods of aflibercept administration), Evans criteria (n ≥ 3, $\chi^2 > 4$, PRR > 2), and $IC_{025} > 0$ for aflibercept were conducted with the Breslow–Day test. †Significance is marked in bold ($p < 0.05$). ‡One or more formulations did not meet the Bonferroni correction threshold, Evans criteria, or $IC_{025} > 0$ for the adverse event and therefore the Breslow–Day test was not conducted. ¶ROR: Not estimable because of a zero count AE in FAERS. AE: adverse event; CI: confidence interval; FAERS: Food and Drug Administration Adverse Event Reporting System; FDA: Food and Drug Administration; HD: high-dose aflibercept (8 mg); IC: information component; PT: preferred term; PRR: proportional reporting ratio; ROR: reporting odds ratio; SD: standard-dose aflibercept (2 mg); SMQ: Standardized MedDRA Queries.

Table S13. MedDRA preferred terms for the ocular AEs mentioned on the package inserts.

Aflibercept 2 mg [SD] Only	Aflibercept 8 mg [HD] Only	Both Formulations
Corneal edema	Eye irritation	Cataract
Foreign body sensation	Retinal hemorrhage	Conjunctival hemorrhage
Injection site pain	Vitreous hemorrhage	Corneal epithelium defect
Lenticular opacities	Retinal pigment epitheliopathy	Endophthalmitis
Scleritis		Eye pain
		Eyelid edema
		Injection site hemorrhage
		Eye inflammation
		Increased intraocular pressure
		Increased lacrimation
		Ocular hyperemia
		Retinal detachment
		Retinal tear
		Retinal vasculitis
		Blurred vision
		Vitreous detachment
		Vitreous floaters
		Detachment of macular retinal pigment epithelium
		Detachment of retinal pigment epithelium
		Hemorrhagic occlusive retinal vasculitis
		Retinal occlusive vasculitis
		Retinal pigment epithelial tear

AE: adverse event; HD: high-dose aflibercept (8 mg); MedDRA: Medical Dictionary for Regulatory Activities; SD: standard-dose aflibercept (2 mg).
